# Constrained Multi-Sensor Control Using a Multi-Target MSE Bound and a δ-GLMB Filter

**DOI:** 10.3390/s18072308

**Published:** 2018-07-16

**Authors:** Feng Lian, Liming Hou, Jing Liu, Chongzhao Han

**Affiliations:** Ministry of Education Key Laboratory for Intelligent Networks and Network Security (MOE KLINNS), School of Electronics and Information Engineering, Xi’an Jiaotong University, Xi’an 710049, China; hliming2017@stu.xjtu.edu.cn (L.H.); elelj20080730@xjtu.edu.cn (J.L.); czhan@xjtu.edu.cn (C.H.)

**Keywords:** multi-sensor control, labeled random finite set, multi-target tracking, error bounds, Bayesian estimation

## Abstract

The existing multi-sensor control algorithms for multi-target tracking (MTT) within the random finite set (RFS) framework are all based on the distributed processing architecture, so the rule of generalized covariance intersection (GCI) has to be used to obtain the multi-sensor posterior density. However, there has still been no reliable basis for setting the normalized fusion weight of each sensor in GCI until now. Therefore, to avoid the GCI rule, the paper proposes a new constrained multi-sensor control algorithm based on the centralized processing architecture. A multi-target mean-square error (MSE) bound defined in our paper is served as cost function and the multi-sensor control commands are just the solutions that minimize the bound. In order to derive the bound by using the generalized information inequality to RFS observation, the error between state set and its estimation is measured by the second-order optimal sub-pattern assignment metric while the multi-target Bayes recursion is performed by using a δ-generalized labeled multi-Bernoulli filter. An additional benefit of our method is that the proposed bound can provide an online indication of the achievable limit for MTT precision after the sensor control. Two suboptimal algorithms, which are mixed penalty function (MPF) method and complex method, are used to reduce the computation cost of solving the constrained optimization problem. Simulation results show that for the constrained multi-sensor control system with different observation performance, our method significantly outperforms the GCI-based Cauchy-Schwarz divergence method in MTT precision. Besides, when the number of sensors is relatively large, the computation time of the MPF and complex methods is much shorter than that of the exhaustive search method at the expense of completely acceptable loss of tracking accuracy.

## 1. Introduction

Sensor control (also known as sensor management) for target tracking [1,2] generally refers to improvement of target detection and estimation accuracy by making single or multiple sensors perform certain operations under some given constraint conditions. The common constraints include the limitations of the communication range and bandwidth, energy consumption, computation cost, collision avoidance, and field of view (FoV) of individual sensor nodes, etc. The common operations include changing the sensor position, velocity or gesture, adjusting sensor work mode, selecting sensor type and number, scheduling sensor observation time, etc. Due to the uncertainty of target number and state, measurement noise, missed detection, clutter, nonlinear, real-time requirements and so on, the sensor control compared with the traditional control is more complicated, and thus attracts widespread attention [3,4,5,6,7].

In recent years, the theory of random finite set (RFS) [8] has been extensively applied in the problem of multi-target tracking (MTT). Especially with the two newest RFS-based MTT methods which are δ-generalized labeled the multi-Bernoulli (δ-GLMB) filter [9,10,11,12,13] and the Poisson multi-Bernoulli mixture (PMBM) filter [14,15], have aroused a great deal of interest because of their conjugacy (also known as the conjugate priors) in common. The conjugacy means exactly that given an initial density, all subsequent predicted and posterior densities have the same form as the initial density. In other words, the families of conjugate priors densities are closed-form in Bayes inference. The conjugacy of the GLMB and PMBM families under the standard multi-target dynamic and observation models is a remarkable advantage in MTT, since the Bayes recursions for non-conjugate RFS densities are usually intractable because of their high computation costs. As a result, both the δ-GLMB and PMBM filters have much better MTT performance than other multi-target RFS filters, such as probability hypothesis density (PHD) [16], cardinality-balanced multi-target multi-Bernoulli (CBMeMBer) [17], and cardinalized PHD (CPHD) [18].

With the rapid development of RFS theory, the RFS-based sensor control methods for MTT have also become a new research hotspot. By the use of RFS, the problem of MTT and sensor control can be unified as a partially observed Markov decision process (POMDP) [19] within the Bayes framework. A recursion of the POMDP for sensor control is: Given the previous sensor control command and observations, the current control command is just the optimal solution of a specified objective function (also called cost or reward function); then the current observations are received by the sensors after executing the control command; finally, the Bayes update and prediction steps are completed.

Based on this, some results [20,21,22,23,24,25,26] have been achieved recently for the single sensor control for MTT. However, the control complexity will increase significantly for the multi-sensor cases. To the best of our knowledge, there are only two methods for the multi-sensor control until now, which are the maximization of Cauchy-Schwarz (CS) divergence in [27] and the minimization of posterior expected error of cardinality and states (PEECS) in [28]. Both methods are based on the distributed processing architecture, so the rule of generalized covariance intersection (GCI) [29,30] has to be used to obtain the multi-sensor posterior density by the fusion of the local posterior densities of all individual sensor nodes. However, so far there has still been no reliable basis for setting the normalized fusion weight of each sensor in GCI. In the two existing methods, the fusion weights of GCI are only determined based on experience. For example, when the sensors have the same or similar observation performance, the weights can be set to the same; conversely, it is difficult to find an effective method to set the weights correctly. In addition, although the commands of the multi-sensor control problem can be found by exhaustive search method, its computation cost will increase significantly with the increase of the number of sensors. The coordinate descent method [31] was applied by Wang et al. [28] to reduce the computation cost by finding a suboptimal multi-sensor control command. But this method can only be used for the unconstrained multi-sensor control problem. In fact, except the single-sensor control algorithm in [26], the other RFS-based sensor control methods do not consider the possible constraint conditions.

In this paper, a new constrained multi-sensor control algorithm is proposed for MTT by performing the multi-target Bayes recursion with the δ-GLMB filter as well as taking the multi-target mean-square error (MSE) bound as the cost function. Since the error here refers to the distance between the multi-target state set and its estimation, it is defined by the second-order optimal sub-pattern assignment (OSPA) metric [32] rather than the traditional Euclidean metric for random vectors. In fact, the former has been widely applied in evaluating the estimation accuracy for MTT algorithms. In order to obtain the lower bound of this error, we firstly need to extend the usual information inequality [33] for measurement vector to RFS observation. The bounds of this paper are conditioned on the specific observation setups to the current moment, which contain useful information about the realization of multi-target states. As a result, it can more accurately indicate the multi-target online estimation performance, and is more suitable for real-time sensor control than the unconditional bounds [34]. The latter are obtained by taking the expectation with regards to the joint density of states and measurements, so that they average out the valuable information about state realization and are only decided by the dynamic and observation models. 

When the number of sensors is relatively large, it is infeasible to find the optimal control commands by using the exhaustive search method due to its very high computation cost. To tackle this, two suboptimal methods, called the mixed penalty function (MPF) method [35] and the complex method [36], are proposed to reduce the calculation burden for solving the constrained optimization problem in the multi-sensor control. At last, our method does not need to adopt the GCI fusion rule since it is performed based on a centralized processing structure.

Finally, the simulation results show that for the constrained multi-sensor control system with distinct observation performance, our method can provide a more effective sensor control strategy than the GCI-based CS divergence method in [27], and so its multi-target estimation accuracy is much better than the latter. Furthermore, compared with the exhaustive search method, both of the MPF and complex methods can significantly reduce the computation time for the constrained sensor control especially in the cases with a large number of sensor nodes at the expense of completely acceptable loss of tracking accuracy.

The current version of the proposed algorithm is based on a centralized processing architecture, so it is appropriate for the environment where the individual sensors have no data processing capabilities and the reliability of communications between the fusion center and each of the sensor nodes is necessary. Because of this, the current algorithm cannot be used directly for a distributed sensor network of geographically dispersed sensor nodes with limited communication, independent data processing capabilities, and no central fusion node. One of our future work will focus on extending the proposed algorithm to a distributed processing architecture.

The rest of this paper is organized as follows. Section 2 describes the problem of constrained multi-sensor control for MTT within the RFS framework. In Section 3, a multi-target MSE bound is obtained as the cost function of multi-sensor control, and then a detailed recursion of our method is summarized. In Section 4, the MPF and complex methods are proposed to reduce the computation cost of our method. Section 5 verifies the effectiveness of our method by two simulation examples. Conclusions and future work are given in Section 6. Mathematical proofs are attached in the Appendix.

## 2. Problem Formulation

First, some necessary instructions on the symbols and functions of this paper are presented as follows.

For easy distinction, we used italics to indicate the unlabeled variables and boldface to indicate the labeled variables. For example, the conventional unlabeled state vector, measurement vector, and their sets are marked as x, z, X, and Z, while the labeled state vector and its set are marked as x and X. Let $X_{n}$, $X_{n}$, and |X| respectively denote a n-element set, the space of $X_{n}$ and the cardinality of X. $\delta_{Y}(X)$, $1_{Y}(X)$, and $p^{X}$ are the generalized functions of Kronecker delta, inclusion indicator, and the multi-target exponential, respectively:

(1) $$\delta_{Y}(X)≜\left\{ \begin{array}{l} \begin{array}{cc} 1, & \mathrm{if}\;X=Y \end{array}\\ \begin{array}{cc} 0, & \mathrm{otherwise} \end{array} \end{array}\right.$$

(2) $$1_{Y}(X)≜\left\{ \begin{array}{l} \begin{array}{cc} 1, & \mathrm{if}\;X\subseteq Y \end{array}\\ \begin{array}{cc} 0, & \mathrm{otherwise} \end{array} \end{array}\right.$$

(3) $$p^{X}≜\left\{ \begin{array}{ll} \prod_{x\in X}p(x), & X\neq \emptyset \\ 1, & X=\emptyset \end{array}\right.$$

where $1_{Y}\left(\left\{x\right\}\right)$ is usually abbreviated as $1_{Y}(x)$.

Let $\hat{x}$ be an unbiased estimate of x from $Z_{m}$ and let $f\left(x,Z_{m}\right)$ be a joint density of $\left(x,Z_{m}\right)$ over the space $X_{1}\times {\mathbb{Z}}_{m}$. Then, assuming that regularity conditions hold and $\partial^{2}\log f\left(x,Z_{m}\right)/\partial x^{i}\partial x^{j}$ exists, the generalized information inequality is obtained by extending the definition of the usual information inequality [33] for random vector to the space of RFS observation,

(4) $$\begin{array}{cc} \int_{{\mathbb{Z}}_{m}}\int_{X_{1}}f\left(x,Z_{m}\right){\left(x^{l}-{\hat{x}}^{l}\right)}^{2}dxdz_{1:m}\geq {\left[J_{m}^{-1}\right]}^{l,l}, & l=1,\ldots ,L \end{array}$$

where $z_{1:m}≜z_{1},\ldots ,z_{m}$, L is the dimension of x, $x^{l}$ and ${\hat{x}}^{l}$ are the lth components of x and $\hat{x}$, and $J_{m}$ is the L×L Fisher information matrix (FIM) conditioned on |Z|=m with the components:

(5) $$\begin{array}{cc} {\left[J_{m}\right]}^{i,j}=-E_{f}\left[\frac{\partial^{2}\log f\left(x,Z_{m}\right)}{\partial x^{i}\partial x^{j}}\right]=-\int_{{\mathbb{Z}}_{m}}\int_{X_{1}}f\left(x,Z_{m}\right)\frac{\partial^{2}\log f\left(x,Z_{m}\right)}{\partial x^{i}\partial x^{j}}dxdz_{1:m}, & i,j=1,\ldots ,L \end{array}$$

According to [33], (4) is satisfied with equality if and only if the density $f\left(x,Z_{m}\right)$ obeys a distribution of exponential family.

Assume that multiple independently moving targets are observed by s independently controllable sensors. Targets may die, survive, or be born over time. Sensors may receive the measurements from targets and clutters, or miss detection. The set X of multi-target states is modeled as a labeled RFS over the space X×L, where X and L respectively represent the state space and discrete label space. The survival probability and transition density of a single-target state x=(x,ℓ)∈X are, respectively, $p_{S}\left(x,\ell \right)$ and $f\left(x,\ell |x^{\prime },\ell^{\prime }\right)\delta_{\ell^{\prime }}(\ell )$, where $x^{\prime }=\left(x^{\prime },\ell^{\prime }\right)$ is the state of the target at the last time.

The set $Z^{i}$ of the ith (i=1,…,s) sensor’s measurements is modeled as a RFS over the space ${\mathbb{Z}}^{i}$. Let $z^{i}\in Z^{i}$ be a single measurement vector and $u^{i}\in U^{i}$ the control command, where $U^{i}$ is the command space. The clutter is modeled as a Poisson RFS with the intensity:

(6) $$\kappa^{i}\left(z^{i}\right)=\lambda^{i}f_{\kappa }^{i}\left(z^{i}\right)$$

where $\lambda^{i}$ and $f_{\kappa }^{i}\left(z^{i}\right)$ are the averaged clutter number per scan and single-clutter density, respectively. According to [8], the likelihood $g^{i}\left(Z^{i}|X,u^{i}\right)$ can be written as:

(7) $$g^{i}\left(Z^{i}|X,u^{i}\right)=e^{-\lambda^{i}}{\left[\kappa^{i}\right]}^{Z^{i}}\sum_{\theta^{i}\in \Theta^{i}}\delta_{{\left(\theta^{i}\right)}^{-1}(\{0:|Z^{i}|\})}\left(L(X)\right){\left[\psi_{Z^{i},u^{i}}^{i}\left(\cdot ;\theta^{i}\right)\right]}^{X}$$

where L(X) is the label set of X, $\theta^{i}$ is the association map $L\to \left\{0:\left|Z^{i}\right|\right\}≜\left\{0,1,\ldots ,\left|Z^{i}\right|\right\}$ and $\Theta^{i}$ is the space of $\theta^{i}$:

(8) $$\psi_{Z^{i},u^{i}}^{i}\left(x,\ell ;\theta^{i}\right)=\delta_{0}\left(\theta^{i}(\ell )\right)\left(1-p_{D}^{i}\left(x,\ell ,u^{i}\right)\right)+\left(1-\delta_{0}\left(\theta^{i}(\ell )\right)\right)\frac{p_{D}^{i}\left(x,\ell ,u^{i}\right)g^{i}\left(z_{\theta^{i}(\ell )}^{i}|x,\ell ,u^{i}\right)}{\kappa^{i}\left(z_{\theta^{i}(\ell )}^{i}\right)}$$

where $p_{D}^{i}\left(x,\ell ,u^{i}\right)$ and $g^{i}\left(z^{i}|x,\ell ,u^{i}\right)$ are, respectively, the detection probability and likelihood of a single target.

In order to simplify the formulas of this paper, we omitted the two cumulative sequences of multi-sensor measurement sets and control commands up to the last time, and used the subscript ‘+’ to indicate the predicted density or variable. Define that $Z^{1:s}≜Z^{1},\ldots ,Z^{s}$ and $u^{1:s}≜u^{1},\ldots ,u^{s}$. Within the Bayes framework, the estimated set $\hat{X}$ of multi-target states from the measurement sets $Z^{1:s}$ of the s independently controllable sensors is obtained by the recursion of the posterior multi-target density $\pi \left(X|Z^{1:s},u^{1:s}\right)$:

(9) $$\pi_{+}(X)=\int f\left(X|X^{\prime }\right)\pi \left(X^{\prime }\right)\delta X^{\prime }$$

(10) $$\pi \left(X|Z^{1:s},u^{1:s}\right)=\frac{\prod_{i=1}^{s}g^{i}\left(Z^{i}|X,u^{i}\right)\pi_{+}(X)}{\int \prod_{i=1}^{s}g^{i}\left(Z^{i}|X,u^{i}\right)\pi_{+}(X)\delta X}$$

where $X^{\prime }$ is the set of multi-target states at the last time, $\pi_{+}(X)$ and $f\left(X|X^{\prime }\right)$ are the predicted and transition densities of the multi-target state set, and ∫⋅δX denotes the integration of a labeled RFS, which is defined by:

(11) $$\int b(X)\delta X=\sum_{n=0}^{\infty }\frac{1}{n!}\sum_{\ell_{1:n}\in L_{n}}\int_{X_{n}}b\left(X_{n}\right)dx_{1:n}$$

where b(X) is a function of X, $x_{1:n}≜x_{1},\ldots ,x_{n}$ and $\ell_{1:n}≜\ell_{1},\ldots ,\ell_{n}$.

The purpose of the constrained multi-sensor control for the Bayes MTT system is to find the optimal multi-sensor control commands ${\left[u^{1:s}\right]}^{*}$ before the measurement sets $Z^{1:s}$ are received, which satisfies:

(12) $$\begin{array}{l} {\left[u^{1:s}\right]}^{*}=\underset{u^{1:s}\in U^{1:s}}{\arg\min/\max}\;\vartheta \left(u^{1:s};\pi_{+}\right)\\ s.t.\left\{ \begin{array}{l} \begin{array}{cc} \gamma_{i}\left(u^{1:s};\pi_{+}\right)\geq 0 & i=1,\ldots ,l \end{array}\\ \begin{array}{cc} \nu_{j}\left(u^{1:s};\pi_{+}\right)=0 & j=1,\ldots ,m \end{array} \end{array}\right. \end{array}$$

where $\vartheta \left(u^{1:s};\pi_{+}\right)$, $\gamma_{i}\left(u^{1:s};\pi_{+}\right)\geq 0$ and $\nu_{j}\left(u^{1:s};\pi_{+}\right)=0$ are the objective function, i is the inequality constraint, and j is the equality constraint of the control commands $u^{1:s}$, given the predicted multi-target density $\pi_{+}(X)$; $u^{1:s}\in U^{1:s}$ denotes $u^{1}\in U^{1},\ldots ,u^{s}\in U^{s}$. The specific forms of $\gamma_{i}\left(u^{1:s};\pi_{+}\right)$ and $\nu_{j}\left(u^{1:s};\pi_{+}\right)$ are usually determined according to the system requirements or prior knowledge. Let:

(13) $$U_{D}^{1:s}=\left\{u^{1:s}\in U^{1:s}|\gamma_{i}\left(u^{1:s};\pi_{+}\right)\geq 0,i=1,\ldots ,l;\nu_{j}\left(u^{1:s};\pi_{+}\right)=0,j=1,\ldots ,m\right\}$$

be the feasible domain of $u^{1:s}$, $U_{D}^{1:s}\subseteq U^{1:s}$.

The current version of this paper only focuses on the multi-sensor one-step ahead control. Our method can be extended to the case of H-step ahead control by replacing the one-step predicted density $\pi_{+}(X)$ with the H-step predicted density $\pi_{+H}(X)$.

## 3. Multi-Sensor Multi-Target MSE Bound

The labeled RFS-based MSE for the multi-target Bayes estimation from the s independently controllable sensors is defined by:

(14) $$\begin{array}{ll} \sigma^{2}\left(u^{1:s}\right) & ≜E\left[e^{2}\left(X,\hat{X}\right)\right]\\ & =\int_{{\mathbb{Z}}^{s}}\cdots \int_{{\mathbb{Z}}^{1}}\int_{X\times L}f\left(X,Z^{1:s}|u^{1:s}\right)e^{2}\left(X,\hat{X}\right)\delta X\delta Z^{1:s}\\ & =\int_{{\mathbb{Z}}^{s}}\cdots \int_{{\mathbb{Z}}^{1}}\int_{X\times L}\prod_{i=1}^{s}g^{i}\left(Z^{i}|X,u^{i}\right)\pi_{+}(X)e^{2}\left(X,\hat{X}\right)\delta X\delta Z^{1:s} \end{array}$$

where $f\left(X,Z^{1:s}|u^{1:s}\right)$ is the joint density of $\left(X,Z^{1:s}\right)$ conditioned on $u^{1:s}$, and $e\left(X,\hat{X}\right)$ is the error between X and $\hat{X}$.

In this paper, the optimal multi-sensor control commands are obtained by minimizing the lower bound ${\underline{\sigma }}^{2}\left(u^{1:s}\right)$ of $\sigma^{2}\left(u^{1:s}\right)$. For simplicity, the predicted multi-target density $\pi_{+}(X)$ is treated as a default condition and no longer explicitly appears in (12). Then, (12) can be rewritten as:

(15) $$\begin{array}{l} {\left[u^{1:s}\right]}^{*}=\underset{u^{1:s}\in U^{1:s}}{\arg\min}{\underline{\sigma }}^{2}\left(u^{1:s}\right)\\ s.t.\left\{ \begin{array}{l} \begin{array}{cc} \gamma_{i}\left(u^{1:s}\right)\geq 0 & i=1,\ldots ,l \end{array}\\ \begin{array}{cc} \nu_{j}\left(u^{1:s}\right)=0 & j=1,\ldots ,m \end{array} \end{array}\right. \end{array}$$

The following three assumptions are given in order to obtain ${\underline{\sigma }}^{2}\left(u^{1:s}\right)$.

**Assumption** **1.** *The error $e\left(X,\hat{X}\right)$ is defined by the second-order OSPA metric [32]:*

(16) $$e\left(X,\hat{X}\right)≜\left\{ \begin{array}{l} \begin{array}{c} 0\;|\hat{X}|=|X|=0 \end{array}\\ {\left(\frac{\underset{\tau \in \Gamma_{\max(|\hat{X}|,|X|)}}{\min}\sum_{i=1}^{\min(|\hat{X}|,|X|)}\min\left(c^{2},{\left\|x_{i}-{\hat{x}}_{\tau_{i}}\right\|}^{2}\right)+c^{2}\mathrm{abs}\left(\left|\hat{X}|-|X\right|\right)}{\max\left(\left|\hat{X}|,|X\right|\right)}\right)}^{\frac{1}{2}}\;|\hat{X}|+|X|>0 \end{array}\right.$$

where $\Gamma_{n}$ is a set of all permutations on {1,…,n} and $\tau =\left\{\tau_{1:n}\right\}$ is an element of $\Gamma_{n}$, c is a cut-off parameter, max(⋅), min(⋅), abs(⋅), and ||⋅|| denote the operations of taking the maximum, minimum, absolute value and 2-norm.

**Assumption** **2.** *The multi-target Bayes recursion is performed by the δ-GLMB filter. So, the predicted multi-target density $\pi_{+}(X)$ is δ-GLMB of the form:*

(17) $$\pi_{+}(X)=\Delta (X)\sum_{(I,\xi )\in F(L)\times \Xi }\delta_{I}\left(L(X)\right)\omega_{+}^{\left(I,\xi \right)}{\left[p_{+}^{\left(\xi \right)}\right]}^{X}$$

where $\Delta (X)≜\delta_{\left|X\right|}\left(\left|L(X)\right|\right)$ is a distinct indicator for the labels of X, I∈F(L) is a track label set in the collection F(L) of finite subsets of L, ξ∈Ξ is a cumulative sequence of multi-sensor association maps up to the last time in the discrete space Ξ, the weight $\omega_{+}^{\left(I,\xi \right)}$ denotes the probability that the track set I has an association history ξ, and $p_{+}^{\left(\xi \right)}(x)$ is the predicted density of a labeled state x given ξ.

According to the conjugacy of the δ-GLMB filter, the predicted and posterior multi-target δ-GLMB densities are closed under the Bayes recursion.

**Assumption** **3.** *Maximum a posterior (MAP) detection and unbiased estimation criteria. The joint multi-target estimator or marginal multi-target estimator [8] can theoretically be used to obtain the optimal estimate of multi-target state set X from the posterior density $\pi \left(X|Z^{1:s},u^{1:s}\right)$. However, both of the estimators are very hard to calculate. Actually, almost all of the multi-target Bayes filters adopt a suboptimal method, where the target number estimate is firstly obtained by using a MAP detector and then the individual state estimates are obtained by using an unbiased estimator based on the estimated target number. So, the two criteria are also adopted in our paper to be consistent with most of multi-target filters.*

**Lemma** **1.** *Given Assumptions 2 and 3, MAP detector determines $|\hat{X}|=\hat{n}$ ($\hat{n}=0,1,\ldots ,\infty$) if and only if $Z^{1:s}\subseteq {\mathbb{Z}}_{\hat{n}}^{1:s}$:*

(18) $${\mathbb{Z}}_{\hat{n}}^{1:s}=\left\{\begin{array}{cc} Z^{1:s}\subseteq {\mathbb{Z}}^{1:s}: & \hat{n}=\underset{n}{\arg\max}\left(\sum_{(I,\xi )\in F_{n}(L)\times \Xi }\sum_{\theta^{1:s}\in \Theta^{1:s}}\omega_{Z^{1:s},u^{1:s}}^{\left(\xi ,\theta^{1:s}\right)}(I)\right) \end{array}\right\}$$

where ${\mathbb{Z}}^{1:s}≜{\mathbb{Z}}^{1}\times \cdots \times {\mathbb{Z}}^{s}$ is the joint measurement space of the s sensors, ${\mathbb{Z}}_{\hat{n}}^{1:s}≜{\mathbb{Z}}_{\hat{n}}^{1}\times \cdots \times {\mathbb{Z}}_{\hat{n}}^{s}$ is the subspace of ${\mathbb{Z}}^{1:s}$ where the target number is estimated as $\hat{n}$, ${\mathbb{Z}}_{0}^{1:s},{\mathbb{Z}}_{1}^{1:s},\ldots ,{\mathbb{Z}}_{\infty }^{1:s}$ constitute a partition of ${\mathbb{Z}}^{1:s}$, $\theta^{1:s}\in \Theta^{1:s}$ denotes $\theta^{1}\in \Theta^{1},\ldots ,\theta^{s}\in \Theta^{s}$, and $F_{n}(L)$ is the collection of n-element subsets of L,

(19) $$\omega_{Z^{1:s},u^{1:s}}^{\left(\xi ,\theta^{1:s}\right)}(I)=\frac{\omega_{+}^{\left(I,\xi \right)}{\langle p_{+}^{\left(\xi \right)}\left(\cdot ,\ell \right),\prod_{i=1}^{s}\delta_{{\left(\theta^{i}\right)}^{-1}(\{0:|Z^{i}|\})}(I)\psi_{Z^{i},u^{i}}^{i}\left(\cdot ,\ell ;\theta^{i}\right)\rangle }^{I}}{\sum_{(I,\xi )\in F(L)\times \mathbb{Z}}\omega_{+}^{\left(I,\xi \right)}\sum_{\theta^{1:s}\in \Theta^{1:s}}{\langle p_{+}^{\left(\xi \right)}\left(\cdot ,\ell \right),\prod_{i=1}^{s}\delta_{{\left(\theta^{i}\right)}^{-1}(\{0:|Z^{i}|\})}(I)\psi_{Z^{i},u^{i}}^{i}\left(\cdot ,\ell ;\theta^{i}\right)\rangle }^{I}}$$

actually indicates the conditional probability that the track label set is I, the association maps up to the last time are ξ, and the current association map is $\theta^{1:s}$, given $Z^{1:s}$ and $u^{1:s}$, the sum $\sum_{(I,\xi )\in F_{n}(L)\times \Xi }\sum_{\theta^{1:s}\in \Theta^{1:s}}\omega_{Z^{1:s},u^{1:s}}^{\left(\xi ,\theta^{1:s}\right)}(I)$ in (18) actually indicates the posterior probability $P\left(|X|=n|Z^{1:s},u^{1:s}\right)$. The detailed derivations of (18) and (19) are given in the proof of Lemma 1, which will be shown in Appendix A.

Define that $Z_{m^{1:s}}^{1:s}≜Z_{m^{1}}^{1},\ldots ,Z_{m^{s}}^{s}$ and ${\mathbb{Z}}_{m^{1:s}}^{1:s}≜{\mathbb{Z}}_{m^{1}}^{1}\times \cdots \times {\mathbb{Z}}_{m^{s}}^{s}$ is the space of $Z_{m^{1:s}}^{1:s}$, where the subscript $m^{i}$ is the number of measurements received by the ith sensor. Let $q\left(X_{n},Z_{m^{1:s}}^{1:s}|u^{1:s}\right)$ be the joint density over the space ${\left(X\times L\right)}_{n}\times {\mathbb{Z}}_{m^{1:s}}^{1:s}$ conditioned on $u^{1:s}$. According to Bayes formula, we get:

(20) $$q\left(X_{n},Z_{m^{1:s}}^{1:s}|u^{1:s}\right)=\frac{1}{\Omega_{n,m^{1:s}}\left(u^{1:s}\right)}\prod_{i=1}^{s}g^{i}\left(Z_{m^{i}}^{i}|X_{n},u^{i}\right)\pi_{+}\left(X_{n}\right)$$

where $\Omega_{n,m^{1:s}}\left(u^{1:s}\right)$ is a normalization factor:

(21) $$\Omega_{n,m^{1:s}}\left(u^{1:s}\right)=\sum_{\ell_{1:n}\in L_{n}}\int_{{\mathbb{Z}}_{m^{s}}^{s}}\cdots \int_{{\mathbb{Z}}_{m^{1}}^{1}}\int_{X_{n}}\prod_{i=1}^{s}g^{i}\left(Z_{m^{i}}^{i}|X_{n},u^{i}\right)\pi_{+}\left(X_{n}\right)dx_{1:n}dz_{1:m^{1}}^{1}\cdots dz_{1:m^{s}}^{s}$$

where $z_{1:m^{i}}^{i}≜z_{1}^{i},\ldots ,z_{m^{i}}^{i}$, i=1,…,s. It can be known from (21) that $\Omega_{n,m^{1:s}}\left(u^{1:s}\right)/m^{1}!\cdots m^{s}!n!$ actually indicates the probability $P\left(|X|=n,|Z^{1}|=m^{1},\ldots ,|Z^{s}|=m^{s}|u^{1:s}\right)$.

Let $\varpi_{\hat{n},n,m^{1:s}}\left(u^{1:s}\right)$ be the integration of $q\left(X_{n},Z_{m^{1:s}}^{1:s}|u^{1:s}\right)$ over the space ${\left(X\times L\right)}_{n}\times {\mathbb{Z}}_{\hat{n},m^{1:s}}^{1:s}$. From (20), we get:

(22) $$\begin{array}{ll} \varpi_{\hat{n},n,m^{1:s}}\left(u^{1:s}\right) & =\sum_{\ell_{1:n}\in L_{n}}\int_{{\mathbb{Z}}_{\hat{n},m^{s}}^{s}}\cdots \int_{{\mathbb{Z}}_{\hat{n},m^{1}}^{1}}\int_{X_{n}}q\left(X_{n},Z_{m^{1:s}}^{1:s}|u^{1:s}\right)dx_{1:n}dz_{1:m^{1}}^{1}\cdots dz_{1:m^{s}}^{s}\\ & =\frac{1}{\Omega_{n,m^{1:s}}\left(u^{1:s}\right)}\sum_{\ell_{1:n}\in L_{n}}\int_{{\mathbb{Z}}_{\hat{n},m^{s}}^{s}}\cdots \int_{{\mathbb{Z}}_{\hat{n},m^{1}}^{1}}\int_{X_{n}}\prod_{i=1}^{s}g^{i}\left(Z_{m^{i}}^{i}|X_{n},u^{i}\right)\pi_{+}\left(X_{n}\right)dx_{1:n}dz_{1:m^{1}}^{1}\cdots dz_{1:m^{s}}^{s} \end{array}$$

where the measurement subspace ${\mathbb{Z}}_{\hat{n},m^{1:s}}^{1:s}≜{\mathbb{Z}}_{\hat{n},m^{1}}^{1}\times \cdots \times {\mathbb{Z}}_{\hat{n},m^{s}}^{s}$ of the s sensors in the integral region can be obtained by Lemma 1. It can be known from (22) that $\Omega_{n,m^{1:s}}\left(u^{1:s}\right)\varpi_{\hat{n},n,m^{1:s}}\left(u^{1:s}\right)/m^{1}!\cdots m^{s}!n!$ actually indicates the probability $P\left(|\hat{X}|=\hat{n},|X|=n,|Z^{1}|=m^{1},\ldots ,|Z^{s}|=m^{s}|u^{1:s}\right)$.

Substituting (7) and (17) into (21) and (22), and then according to Lemma 12 of [9], $\Omega_{n,m^{1:s}}\left(u^{1:s}\right)$ and $\varpi_{\hat{n},n,m^{1:s}}\left(u^{1:s}\right)$ are finally obtained as:

(23) $$\Omega_{n,m^{1:s}}\left(u^{1:s}\right)=n!e^{-\sum_{i=1}^{s}\lambda^{i}}\prod_{i=1}^{s}{\left[\lambda^{i}\right]}^{m^{i}}\sum_{(I,\xi )\in F_{n}(L)\times \Xi }\omega_{+}^{\left(I,\xi \right)}\sum_{\theta^{1:s}\in \Theta^{1:s}}{\langle p_{+}^{\left(\xi \right)}\left(\cdot ,\ell \right),\prod_{i=1}^{s}\delta_{{\left(\theta^{i}\right)}^{-1}(\{0:m^{i}\})}(I)\varphi_{u^{i}}^{i}\left(\cdot ,\ell ;\theta^{i}\right)\rangle }^{I}$$

(24) $$\varpi_{\hat{n},n,m^{1:s}}\left(u^{1:s}\right)=\frac{n!e^{-\sum_{i=1}^{s}\lambda^{i}}}{\Omega_{n,m^{1:s}}\left(u^{1:s}\right)}\prod_{i=1}^{s}{\left[\alpha_{\hat{n}}^{i}\lambda^{i}\right]}^{m^{i}}\sum_{(I,\xi )\in F_{n}(L)\times \Xi }\omega_{+}^{\left(I,\xi \right)}\sum_{\theta^{1:s}\in \Theta^{1:s}}{\langle p_{+}^{\left(\xi \right)}\left(\cdot ,\ell \right),\prod_{i=1}^{s}\delta_{{\left(\theta^{i}\right)}^{-1}(\{0:m^{i}\})}(I)\phi_{\hat{n},u^{i}}^{i}\left(\cdot ,\ell ;\theta^{i}\right)\rangle }^{I}$$

where:

(25) $$\varphi_{u^{i}}^{i}\left(x,\ell ;\theta^{i}\right)=\delta_{0}\left(\theta^{i}(\ell )\right)\left(1-p_{D}^{i}\left(x,\ell ,u^{i}\right)\right)+\left(1-\delta_{0}\left(\theta^{i}(\ell )\right)\right)\frac{p_{D}^{i}\left(x,\ell ,u^{i}\right)}{\lambda^{i}}$$

(26) $$\phi_{\hat{n},u^{i}}^{i}\left(x,\ell ;\theta^{i}\right)=\delta_{0}\left(\theta^{i}(\ell )\right)\left(1-p_{D}^{i}\left(x,\ell ,u^{i}\right)\right)+\left(1-\delta_{0}\left(\theta^{i}(\ell )\right)\right)\frac{\beta_{\hat{n}}^{i}p_{D}^{i}\left(x,\ell ,u^{i}\right)}{\alpha_{\hat{n}}^{i}\lambda^{i}}$$

(27) $$\alpha_{\hat{n}}^{i}=\int_{{\mathbb{Z}}_{\hat{n},1}^{i}}f_{\kappa }^{i}\left(z^{i}\right)dz^{i}$$

(28) $$\beta_{\hat{n}}^{i}=\int_{{\mathbb{Z}}_{\hat{n},1}^{i}}g^{i}\left(z^{i}|x,\ell ,u^{i}\right)dz^{i}$$

Because $q\left(X_{n},Z_{m^{1:s}}^{1:s}|u^{1:s}\right)$ is permutation invariant over $x_{1:n}$, its marginal density over any of $x_{1:n}$ is the same and can be derived by:

(29) $$q_{n}\left(x,Z_{m^{1:s}}^{1:s}|u^{1:s}\right)=\int_{X_{n-1}}q\left(\left\{x,x_{2:n}\right\},Z_{m^{1:s}}^{1:s}|u^{1:s}\right)dx_{2:n}$$

Substituting (20) into (29), and then according to (17) and the identical equation $\delta_{n}\left(\left|\left\{\ell ,\ell_{2:n}\right\}\right|\right)=\delta_{n-1}\left(\left|\left\{\ell_{2:n}\right\}\right|\right)\left(1-1_{\left\{\ell_{2:n}\right\}}(\ell )\right)$, $q_{n}\left(x,Z_{m^{1:s}}^{1:s}|u^{1:s}\right)$ can be written as:

(30) $$\begin{array}{c} q_{n}\left(x,Z_{m^{1:s}}^{1:s}|u^{1:s}\right)=\frac{1}{\Omega_{n,m^{1:s}}\left(u^{1:s}\right)}\sum_{\ell_{2:n}\in L_{n-1}}\delta_{n-1}\left(\left|\left\{\ell_{2:n}\right\}\right|\right)\left(1-1_{\left\{\ell_{2:n}\right\}}(\ell )\right)\sum_{(I,\xi )\in F_{n}(L)\times \Xi }\omega_{+}^{\left(I,\xi \right)}\delta_{I}\left(\left\{\ell ,\ell_{2:n}\right\}\right)\\ \cdot \int_{X_{n-1}}\prod_{i=1}^{s}g^{i}\left(Z_{m^{i}}^{i}|\left\{x,x_{2:n}\right\},u^{i}\right)p_{+}^{\left(\xi \right)}(x)\prod_{t=2}^{n}p_{+}^{\left(\xi \right)}\left(x_{t}\right)dx_{2:n} \end{array}$$

Suppose that r out of s sensors receive the measurement arising from state x and their indices are marked as $i_{1}\neq ,\ldots ,\neq i_{r}$, while the indices for the other sensors are marked as $i_{1+r}\neq ,\ldots ,\neq i_{s}$, r=0,1,…,s. Substituting (7) into (30) and then simplifying the result, we get:

(31) $$\begin{array}{l} q_{n}\left(x,\ell ,Z_{m^{1:s}}^{1:s}|u^{1:s}\right)\\ =\frac{1}{\Omega_{n,m^{1:s}}\left(u^{1:s}\right)}e^{-\sum_{i=1}^{s}\lambda^{i}}\prod_{i=1}^{s}{\left[\kappa^{i}\right]}^{Z^{i}}\sum_{(I,\xi )\in F_{n}(L)\times \Xi }1_{I}(\ell )\omega_{+}^{\left(I,\xi \right)}p_{+}^{\left(\xi \right)}(x,\ell )\sum_{\theta^{1:s}\in \Theta^{1:s}}\sum_{0\leq i_{1}\neq ,\ldots ,\neq i_{r}\leq s}\sum_{z^{i_{1}}\in Z_{m^{i_{1}}}^{i_{1}}}\cdots \sum_{z^{i_{r}}\in Z_{m^{i_{r}}}^{i_{r}}}\\ \prod_{j=1}^{r}\delta_{{\left(\theta^{i_{j}}\right)}^{-1}(\{0:m^{i_{j}}-1\})}\left(I-\{\ell \}\right)p_{D}^{i_{j}}\left(x,\ell ,u^{i_{j}}\right)g^{i_{j}}\left(z^{i_{j}}|x,\ell ,u^{i_{j}}\right)\cdot \prod_{j=1+r}^{s}\delta_{{\left(\theta^{i_{j}}\right)}^{-1}(\{0:m^{i_{j}}\})}\left(I-\{\ell \}\right)\left(1-p_{D}^{i_{j}}\left(x,\ell ,u^{i_{j}}\right)\right)\\ \cdot {\langle p_{+}^{\left(\xi \right)}\left(\cdot ,\ell \right),\prod_{j=1}^{r}\psi_{Z_{m^{i_{j}}}^{i_{j}}-\{z^{i_{j}}\},u^{i_{j}}}^{i_{j}}\left(\cdot ,\ell ;\theta^{i_{j}}\right)\cdot \prod_{j=1+r}^{s}\psi_{Z_{m^{i_{j}}}^{i_{j}},u^{i_{j}}}^{i_{j}}\left(\cdot ,\ell ;\theta^{i_{j}}\right)\rangle }^{I-\{\ell \}} \end{array}$$

where $\theta^{i_{j}}$ is the association map $L_{n-1}\to \left\{0:m^{i_{j}}-1\right\}$ for j=1,…,r or $L_{n-1}\to \left\{0:m^{i_{j}}\right\}$ for j=1+r,…,s.

**Theorem** **1.** *Given Assumptions 1–3, the lower bound for the multi-sensor multi-target MSE in (14) is obtained as:*

(32) $${\underline{\sigma }}^{2}\left(u^{1:s}\right)=\sum_{m^{s}=0}^{\infty }\cdots \sum_{m^{1}=0}^{\infty }\sum_{n=0}^{\infty }\sum_{\hat{n}=0,n+\hat{n}>0}^{\infty }\frac{\Omega_{n,m^{1:s}}\left(u^{1:s}\right)\varpi_{\hat{n},n,m^{1:s}}\left(u^{1:s}\right)}{m^{1}!\cdots m^{s}!n!}\left(\varepsilon_{\hat{n},n}\Phi_{\hat{n},n,m^{1:s}}\left(u^{1:s}\right)+(1-\varepsilon_{\hat{n},n})c^{2}\right)$$

where c is the cut-off of OSPA, $\Omega_{n,m^{1:s}}\left(u^{1:s}\right)$ and $\varpi_{\hat{n},n,m^{1:s}}\left(u^{1:s}\right)$ are given by (23) and (24):

(33) $$\Phi_{\hat{n},n,m^{1:s}}\left(u^{1:s}\right)=\min\left(c^{2},\frac{1}{\varpi_{\hat{n},n,m^{1:s}}\left(u^{1:s}\right)}\sum_{l=1}^{L}{\left[J_{\hat{n},n,m^{1:s}}^{-1}\left(u^{1:s}\right)\right]}^{l,l}\right)$$

(34) $$\varepsilon_{\hat{n},n}=\frac{\min\left(\hat{n},n\right)}{\max\left(\hat{n},n\right)}$$

where L is the dimension of the unlabeled state x, $J_{\hat{n},n,m^{1:s}}\left(u^{1:s}\right)$ is the L×L FIM conditioned on $\left(|\hat{X}|=\hat{n},|X|=n,|Z^{1}|=m^{1},\ldots ,|Z^{s}|=m^{s}\right)$:

(35) $${\left[J_{\hat{n},n,m^{1:s}}\left(u^{1:s}\right)\right]}^{i,j}=\frac{-1}{\varpi_{\hat{n},n,m^{1:s}}^{2}\left(u^{1:s}\right)}\int_{{\mathbb{Z}}_{\hat{n},m^{s}}^{s}}\cdots \int_{{\mathbb{Z}}_{\hat{n},m^{1}}^{1}}\int_{X_{1}}q_{n}\left(x,Z_{m^{1:s}}^{1:s}|u^{1:s}\right)\frac{\partial^{2}\log q_{n}\left(x,Z_{m^{1:s}}^{1:s}|u^{1:s}\right)}{\partial x^{i}\partial x^{j}}dxdz_{1:m^{1}}^{1}\cdots dz_{1:m^{s}}^{s}\begin{array}{c} \begin{array}{cc} & i,j=1,\ldots ,L \end{array} \end{array}$$

where we set $J_{\hat{n},n,m^{1:s}}\left(u^{1:s}\right)=\infty$ if ${\mathbb{Z}}_{\hat{n},m^{1}}^{1}\cup \cdots \cup {\mathbb{Z}}_{\hat{n},m^{s}}^{s}=\emptyset$, $\hat{n}=0,1,\ldots ,\infty$; $q_{n}\left(x,Z_{m^{1:s}}^{1:s}|u^{1:s}\right)$ is the unlabeled version of (31):

(36) $$q_{n}\left(x,Z_{m^{1:s}}^{1:s}|u^{1:s}\right)=\sum_{\ell \in L_{1}}q_{n}\left(x,\ell ,Z_{m^{1:s}}^{1:s}|u^{1:s}\right)$$

The proof of Theorem 1 is shown in Appendix B.

**Remark** **1.** *The subscript $\hat{n}$ in (32)–(35) only represents an index for possible target number estimates. In other words, the exact number of the estimated targets does not need to be used in the derivation of the bound of our paper. In fact, it can be seen from Theorem 1 and Lemma 1 that given the predicted multi-target density $\pi_{+}(X)$ in (17), the proposed bound ${\underline{\sigma }}^{2}\left(u^{1:s}\right)$ is just determined by sensor likelihoods and so, is completely independent of current specific measurements $Z^{1:s}$. To go further, although the Bayes recursion of multi-target densities can be performed by the δ-GLMB filter with centralized or distributed fusion architecture, the derivation of ${\underline{\sigma }}^{2}\left(u^{1:s}\right)$ for multi-sensor control is really not affected by the fusion architecture once $\pi_{+}(X)$ is given.*

**Remark** **2.** *The maximum numbers for the possible true targets, estimated targets and measurements of each sensor over a surveillance region can usually be preset by prior knowledge. As a result, the infinite terms involved in the summations of (32) reduce to the finite terms.*

**Remark** **3.** *It can be seen from (24) and (35) that the calculation formulas of $\varpi_{\hat{n},n,m^{1:s}}\left(u^{1:s}\right)$ and $J_{\hat{n},n,m^{1:s}}\left(u^{1:s}\right)$ contain the integrations over the measurement subspace ${\mathbb{Z}}_{\hat{n},m^{1:s}}^{1:s}$, and it is difficult to obtain the analytic expressions for them. Therefore, the methods of numerical integration [37] have to be used for calculation of $\varpi_{\hat{n},n,m^{1:s}}\left(u^{1:s}\right)$ and $J_{\hat{n},n,m^{1:s}}\left(u^{1:s}\right)$. To reduce the computation cost, a very efficient numerical integration method, called quasi Monte Carlo (MC) method [38], is applied here while the predicted ideal measurement sets (PIMS) [8,25] are selected as the samples over ${\mathbb{Z}}_{\hat{n},m^{1:s}}^{1:s}$ in this method.*

Finally, Table 1 shows a detailed recursion for the proposed algorithm of constrained multi-sensor control and MTT.

## 4. Optimization for Constrained Multi-Sensor Control

When the number of sensors is small and the command space $U^{1:s}$ is discrete, the exhaustive search method can be used to find the optimal solution for the constrained multi-sensor control problem described in (12) or (15). But its computation cost will increase rapidly with the increase of the number of sensors. To avoid this as much as possible, two alternative methods, called the MPF method [35] and complex method [36], are proposed to calculate the suboptimal solution of (15).

**MPF method:** The constrained optimization in (15) can be relaxed to the corresponding unconstrained optimization by constructing the augmented objective function:

(37) $$F\left(u^{1:s},r\right)={\underline{\sigma }}^{2}\left(u^{1:s}\right)+r\sum_{i=1}^{l}\gamma_{i}^{-1}\left(u^{1:s}\right)+\frac{1}{\sqrt{r}}\sum_{j=1}^{m}\nu_{j}^{2}\left(u^{1:s}\right)$$

where r>0 is a barrier factor, the first penalty term $\sum_{i=1}^{l}\gamma_{i}^{-1}\left(u^{1:s}\right)$ is to restrict the search into the area determined by inequality constraints, the second penalty term $\sum_{j=1}^{m}\nu_{j}^{2}\left(u^{1:s}\right)$ is to force the search to approach the area determined by equality constraints.

Then, all unconstrained optimization methods can be used to solve the relaxed problem. The classic coordinate descent method [31] for unconstrained optimization is chosen here. Table 2 shows the main steps for the MPF method of this paper.

In the MPF method, the improper initial barrier factor r can cause the penalty function to become ill-conditioned. Such that the relaxed unconstrained optimization is rather difficult to be calculated. In order to increase the probability to converge to the global optimum and speed up convergence rate for the MPF method, the initial control command $u_{\left(0\right)}^{1:s}$, the initial barrier factor r and reduction coefficient C can be appropriately selected by means of the rules proposed in [35].

**Complex method:** This method is suitable for the situations with only inequality constraints. Let $\vec{u}={\left[{\left[u^{1}\right]}^{T},\ldots ,{\left[u^{s}\right]}^{T}\right]}^{T}$ be the total vector of control commands for the s sensors, ${\vec{U}}_{D}$ be the feasible domain of $\vec{u}$, N be the dimension of $\vec{u}$. Table 3 shows the main steps for the complex method of this paper.

In Table 3, the initial reflection coefficient t is generally first taken as t=1.3, and in order to avoid dimensionality reduction, the vertexes ${\vec{u}}_{1},\ldots ,{\vec{u}}_{k}$ of the initial complex can be selected in terms of the rules proposed in [36]. For increasing the probability to converge to the global optimum for the complex method, Krus et al. [39] designed an improved computation formula for the reflecting vertex ${\vec{u}}_{R}$. The advantages of the new formula are that it can make ${\vec{u}}_{R}$ move close to the best vertex ${\vec{u}}_{B}$ gradually and may help ${\vec{u}}_{R}$ to jump out of the local optima by introducing a random noise item. However, this method will increase some convergence time. Due to space limitations, the detail for this is not presented here and it can be found in [39].

## 5. Simulations

### 5.1. Example 1: Scenarios with a Small Number of Sensors

In a two-dimensional area S=[−50m,50m]×[−50m,50m], multiple targets are observed by s=4 position-controllable sensors. The observation period is T=30 time steps. The single-target state is noted as x=(x,ℓ) with label $\ell =\left(k_{B},i_{B}\right)$, where $k_{B}$ is the birth time of the target and $i_{B}$ is the index for distinguishing the birth targets at the same time. The unlabeled state is noted as $x={\left[p_{x},{\dot{p}}_{x},p_{y},{\dot{p}}_{y},w\right]}^{T}$, where $\left(p_{x},p_{y}\right)$ and $\left({\dot{p}}_{x},{\dot{p}}_{y}\right)$ are the positions and velocities in X and Y coordinates and w is the turn rate. The dynamic of each target is dominated by the coordinated turn model [40] with the Gaussian transition density:

(38) $$f\left(x,\ell |x^{\prime },\ell^{\prime }\right)=N\left(x;u(x^{\prime }),Q\right)\delta_{\ell^{\prime }}(\ell )$$

where u(⋅) and Q are the transition function for unlabeled state and covariance matrix for process noise:

(39) $$u(x)=\left[\begin{array}{c} p_{x}+{\dot{p}}_{x}\frac{\sin(w\Delta )}{w}-{\dot{p}}_{y}\frac{1-\cos(w\Delta )}{w}\\ {\dot{p}}_{x}\cos(w\Delta )-{\dot{p}}_{y}\sin(w\Delta )\\ p_{y}+{\dot{p}}_{x}\frac{1-\cos(w\Delta )}{w}+{\dot{p}}_{y}\frac{\sin(w\Delta )}{w}\\ {\dot{p}}_{x}\sin(w\Delta )+{\dot{p}}_{y}\cos(w\Delta )\\ we^{-\Delta /\tau_{w}} \end{array}\right]$$

(40) $$Q=\left[\begin{array}{ccccc} \frac{\Delta^{4}}{4}q_{x}^{2} & \frac{\Delta^{3}}{2}q_{x}^{2}\\ \frac{\Delta^{3}}{2}q_{x}^{2} & \frac{\Delta^{2}}{2}q_{x}^{2}\\ & & \frac{\Delta^{4}}{4}q_{y}^{2} & \frac{\Delta^{3}}{2}q_{y}^{2} & \\ & & \frac{\Delta^{3}}{2}q_{y}^{2} & \frac{\Delta^{2}}{2}q_{y}^{2} & \\ & & & & q_{w}^{2} \end{array}\right]$$

where Δ is the sampling interval, $\tau_{w}$ is the time correlation constant of turn rate w, $q_{x}$, and $q_{y}$ are the accelerations in X and Y coordinates, and $q_{w}$ is the noise standard deviation of turn rate w. In this example, we set Δ=1 s, $\tau_{w}=20\;s$, $q_{x}=0.1{\;m/s}^{2}$, $q_{y}=0.05{\;m/s}^{2}$ and $q_{w}=0.01\;\mathrm{rad}/s$. The survival probability is set as $p_{S}(x,\ell )=0.95$.

The set of new-birth targets is modeled as a labeled Poisson RFS with the intensity:

(41) $$D_{B}(x)=\sum_{i=1}^{5}0.04N\left(x;x_{B,i},Q_{B}\right)$$

where the unlabeled new-birth states are assumed to follow the Gaussian distributions with mean $x_{B,i}$ and covariance matrix $Q_{B}$. In this example, we set $x_{B,1}={\left[30\;m,1\;m/s,-30\;m,2\;m/s,0.2\;\mathrm{rad}/s\right]}^{T}$, $x_{B,2}={\left[30\;m,-5\;m/s,30\;m,-3\;m/s,0.2\;\mathrm{rad}/s\right]}^{T}$, $x_{B,3}={\left[-30\;m,-3\;m/s,-30\;m,4\;m/s,-0.3\;\mathrm{rad}/s\right]}^{T}$, $x_{B,4}={\left[-30\;m,1\;m/s,30\;m,-3\;m/s,0.1\;\mathrm{rad}/s\right]}^{T}$, $x_{B,5}={\left[0\;m,-4\;m/s,0\;m,4\;m/s,-0.3\;\mathrm{rad}/s\right]}^{T}$, $Q_{B}=diag(25{\;m}^{2},0.1\;m^{2}{/s}^{2},25{\;m}^{2},0.1\;m^{2}{/s}^{2},0.01\;{\mathrm{rad}}^{2}{/s}^{2})$ and diag(⋅) denotes a diagonal matrix.

The initial sensor positions are set as $u_{0}^{1}={\left[45\;m,45\;m\right]}^{T}$, $u_{0}^{2}={\left[-45\;m,-45\;m\right]}^{T}$, $u_{0}^{3}={\left[45\;m,-45\;m\right]}^{T}$, $u_{0}^{4}={\left[-45\;m,45\;m\right]}^{T}$. Given the ith sensor’s position ${u^{\prime }}^{i}={\left[{p^{\prime }}_{x,u}^{i},{p^{\prime }}_{y,u}^{i}\right]}^{T}$ at the last time, after executing a control command its possible positions at the current time can be described as a set:

(42) $$U^{i}=\left\{\begin{array}{cc} {\left[{p^{\prime }}_{x,u}^{i}+j\rho_{0}\cos\left(k\frac{2\pi }{N_{\theta }}\right),{p^{\prime }}_{y,u}^{i}+j\rho_{0}\sin\left(k\frac{2\pi }{N_{\theta }}\right)\right]}^{T}; & j=0,\ldots ,N_{\rho };k=1,\ldots ,N_{\theta } \end{array}\right\}$$

where $N_{\rho }=2$, $N_{\theta }=8$ and $\rho_{0}=5\;m$. (42) implies that each sensor has 17 possible positions under a control command. The proposed bound is set as ∞ if one of the sensors moves out of the region S.

The single-target likelihood of each sensor is assumed to be the Gaussian density

(43) $$\begin{array}{cc} g^{i}\left(z^{i}|x,\ell ,u^{i}\right)=N\left(z^{i};h^{i}\left(x,u^{i}\right),R^{i}\left(x,u^{i}\right)\right) & i=1,\ldots ,4 \end{array}$$

where $h^{i}\left(x,u^{i}\right)$ and $R^{i}\left(x,u^{i}\right)$ are the observation function and covariance matrix for measurement noise. Both of them are the functions of unlabeled state x and control command $u^{i}$,

(44) $$\left\{ \begin{array}{l} \begin{array}{cc} h^{i}\left(x,u^{i}\right)={\left[\rho^{i}\left(x,u^{i}\right),{ο}^{i}\left(x,u^{i}\right)\right]}^{T},R^{i}\left(x,u^{i}\right)=\mathrm{diag}\left({\left[\varsigma_{\rho }^{i}\left(x,u^{i}\right)\right]}^{2},{\left[\varsigma_{ο}^{i}\left(x,u^{i}\right)\right]}^{2}\right) & i=1,2 \end{array}\\ h^{3}\left(x,u^{3}\right)=\rho^{3}\left(x,u^{3}\right),R^{3}\left(x,u^{3}\right)={\left[\varsigma_{\rho }^{3}\left(x,u^{3}\right)\right]}^{2}\\ h^{4}\left(x,u^{4}\right)={ο}^{4}\left(x,u^{4}\right),R^{4}\left(x,u^{4}\right)={\left[\varsigma_{ο}^{4}\left(x,u^{4}\right)\right]}^{2} \end{array}\right.$$

where $\rho^{i}\left(x,u^{i}\right)$ and ${ο}^{i}\left(x,u^{i}\right)$ are the distance and angle between state x and the ith sensor, $\varsigma_{\rho }^{i}\left(x,u^{i}\right)$ and $\varsigma_{ο}^{i}\left(x,u^{i}\right)$ are their measurement noise standard deviation:

(45) $$\left\{ \begin{array}{l} \begin{array}{cc} \rho^{i}\left(x,u^{i}\right)=\left\|{\left[x(1,1),x(3,1)\right]}^{T}-u^{i}\right\| & i=1,2,3 \end{array}\\ \begin{array}{cc} {ο}^{i}\left(x,u^{i}\right)=\arctan\frac{x(3,1)-p_{y,u}^{i}}{x(1,1)-p_{x,u}^{i}} & i=1,2,4 \end{array}\\ \begin{array}{cc} \varsigma_{\rho }^{i}\left(x,u^{i}\right)=\varsigma_{0,\rho }^{i}+\eta_{\rho }^{i}\rho^{i}\left(x,u^{i}\right) & i=1,2,3 \end{array}\\ \begin{array}{cc} \varsigma_{ο}^{i}\left(x,u^{i}\right)=\varsigma_{0,ο}^{i}+\eta_{ο}^{i}\rho^{i}\left(x,u^{i}\right) & i=1,2,4 \end{array} \end{array}\right.$$

where $\left(\varsigma_{0,\rho }^{i},\varsigma_{0,ο}^{i}\right)$ and $\left(\eta_{\rho }^{i},\eta_{ο}^{i}\right)$ are the zero-distance measurement noise standard deviations and their increment rates along with the target-sensor distance for the ith sensor. In this example, we set $\varsigma_{0,\rho }^{1}=1\;m$, $\eta_{\rho }^{1}=0.1$, $\varsigma_{0,ο}^{1}=0.02\;\mathrm{rad}$, $\eta_{ο}^{1}=0.002\;\mathrm{rad}/m$; $\varsigma_{0,\rho }^{2}=5\;m$, $\eta_{\rho }^{2}=0.02$, $\varsigma_{0,ο}^{2}=0.1\;\mathrm{rad}$, $\eta_{ο}^{2}=0.0004\;\mathrm{rad}/m$; $\varsigma_{0,\rho }^{3}=2.5\;m$, $\eta_{\rho }^{3}=0.05$; $\varsigma_{0,ο}^{4}=0.05\;\mathrm{rad}$, and $\eta_{ο}^{4}=0.001\;\mathrm{rad}/m$.

The detection probability for each sensor is:

(46) $$\begin{array}{cc} p_{D}^{i}\left(x,\ell ,u^{i}\right)=p_{0,D}^{i}\left(1-\eta_{D}^{i}\rho^{i}\left(x,u^{i}\right)\right) & i=1,\ldots ,4 \end{array}$$

where $p_{0,D}^{i}$ and $\eta_{D}^{i}$ are the zero-distance detection probability and its decrement rate along with the target-sensor distance for the ith sensor. In this example, we set $p_{0,D}^{1}=0.98$, $\eta_{D}^{1}=0.007{\;m}^{-1}$; $p_{0,D}^{2}=0.8$, $\eta_{D}^{2}=0.001{\;m}^{-1}$; $p_{0,D}^{3}=0.92$, $\eta_{D}^{3}=0.005{\;m}^{-1}$; $p_{0,D}^{4}=0.86$, and $\eta_{D}^{4}=0.003{\;m}^{-1}$.

The Poisson clutter intensity for each sensor is:

(47) $$\begin{array}{cc} \kappa^{i}\left(z^{i}\right)=\lambda^{i}U\left(z^{i};S\right) & i=1,\ldots ,4 \end{array}$$

where U(⋅;S) is the uniform density over the area S. In this example, we set $\lambda^{1}=50$, $\lambda^{2}=40$, $\lambda^{3}=30$, $\lambda^{4}=20$.

Suppose that the motion of each sensor needs to consume the energy, the amount of which is proportional to its motion distance. It is required that the total amount of energy consumed by all sensors for each control must not exceed the threshold E. So the inequality constraint model for energy consumption can be written as:

(48) $$\gamma_{1}\left(u^{1:s}\right)=E-\sum_{i=1}^{s}\chi^{i}\left\|u^{i}-{u^{\prime }}^{i}\right\|\geq 0$$

where $\chi^{i}$ is the amount of consumed energy per meter for the motion of the ith sensor. In this example, we set E=100 J, $\chi^{1}=10\;J/m$, $\chi^{2}=8\;J/m$, $\chi^{3}=6\;J/m$, $\chi^{4}=4\;J/m$.

In order to avoid the collision caused by sensor or target, it is required that the distance between any two sensors or between any sensor and any target should not be less than the respective collision avoidance thresholds $T_{1}$ and $T_{2}$. Moreover, since the true target number and positons are unknown, here they are replaced by their predictions. Finally, the inequality constraint models for collision avoidance can be written as:

(49) $$\gamma_{2}\left(u^{1:s}\right)=\underset{1\leq i\neq j\leq s}{\min}\left\|u^{i}-u^{j}\right\|-T_{1}\geq 0$$

(50) $$\gamma_{3}\left(u^{1:s}\right)=\underset{1\leq i\leq s,1\leq j\leq {\hat{n}}_{+}}{\min}\left\|u^{i}-{\left[{\hat{x}}_{+,j}(1,1),{\hat{x}}_{+,j}(3,1)\right]}^{T}\right\|-T_{2}\geq 0$$

where the predicted target number ${\hat{n}}_{+}$ and the unlabeled state ${\hat{x}}_{+,j}$ ($j=1,\ldots ,{\hat{n}}_{+}$) can be extracted from the predicted multi-target density $\pi_{+}(X)$ in (17). In this example, we set $T_{1}=T_{2}=5\;m$.

From the above sensor performance parameters, it can be seen that:

(1) Sensors 1 and 2 can receive the distance and angle measurements of a target. That is, the target position is completely observable for the two sensors. The zero-distance observation accuracy and detection probability of Sensor 1 are the highest, but the observation accuracy and detection probability decrease the most quickly with the increase of the target-sensor distance. Sensor 2 is just the opposite to Sensor 1.

(2) Sensor 3 or 4 can only receive the distance or angle measurement of a target. That is, the target position is partially observable for the two sensors. Their zero-distance observation accuracy and detection probability are medium, also for their decrement rates.

(3) The clutter density and energy consumption per meter of Sensor 1 are the highest, and the corresponding parameters to Sensors 2, 3, and 4 decrease successively from high to low.

Above all, we firstly obtained an intuitive conclusion: How to effectively control Sensor 1 so that it can move as close as possible to the survival targets is the most important factor to improve the MTT accuracy. In other words, Sensor 1 should have the highest control priority compared with the other sensors. Next, we verify the conclusion by simulations.

In this example, the maximum numbers for the true targets, estimated targets, and measurements of every sensor were set as 10, 10, and 100, respectively; the cut-off parameter of OSPA was set as c=100. A particle filter [41] was used to implement our methods.

According to the objective functions and optimization methods for control commands, we abbreviated the algorithms to be compared as: CS divergence with exhaustive search, error bound with exhaustive search, error bound with MPF, and error bound with complex. The first algorithm is proposed in [25], while the last three algorithms are proposed in this paper. As in [27] and [28], the GCI weights used in the CS divergence with exhaustive search were set to the same. By convention, the random control method was still chosen as a standard comparison object to verify the effectiveness of other sensor control algorithms. Here, the random control method was to specify the sensor positions at each time step by randomly selecting j and k in (42) under the constraint conditions of (48)–(50). Obviously, a well-designed sensor control algorithm is clearly useless if its performance is even inferior to that of the random control method. All algorithms are coded by use of the software MATLAB R2018a. The desktop computer used for testing these algorithms was a Lenovo A8800F with the central processing unit (CPU) of an Intel core i7-8700k@3.7–4.7 GHz and 32 GB random access memory (RAM).

In this paper, only the algorithm of CS divergence proposed outside of this paper was used in the comparison. There were two main reasons for this:

(1) So far, there are only two multi-sensor control methods for MTT within the RFS framework: the CS divergence based algorithm [27] and the PEECS-based algorithm [28]. Although the two algorithms have different objective functions for multi-sensor control, their performance is very similar since both methods are based on the distributed processing architecture and have to apply the GCI rule to obtain the multi-sensor posterior density. Furthermore, the method of CS divergence may slightly outperform the method of PEECS in some certain circumstances because the latter applies an approximated δ-GLMB filter called LMB filter in each sensor node. The simulations in Reference [30] have shown that the performance of the LMB filter will decline dramatically in the cases of low signal-to-noise ratio (SNR).

(2) The existing multi-sensor control methods for MTT without the RFS framework [3,4,5,6,7] were not suitable for comparison with our algorithm since almost all of them implicitly assume that the data association between targets and measurements has been completed and the number of targets is known before the sensor control.

As a result, in our opinion, the algorithm of CS divergence in [27] was the best choice to be used in the comparison with the algorithms proposed in this paper.

**Figure 1:**
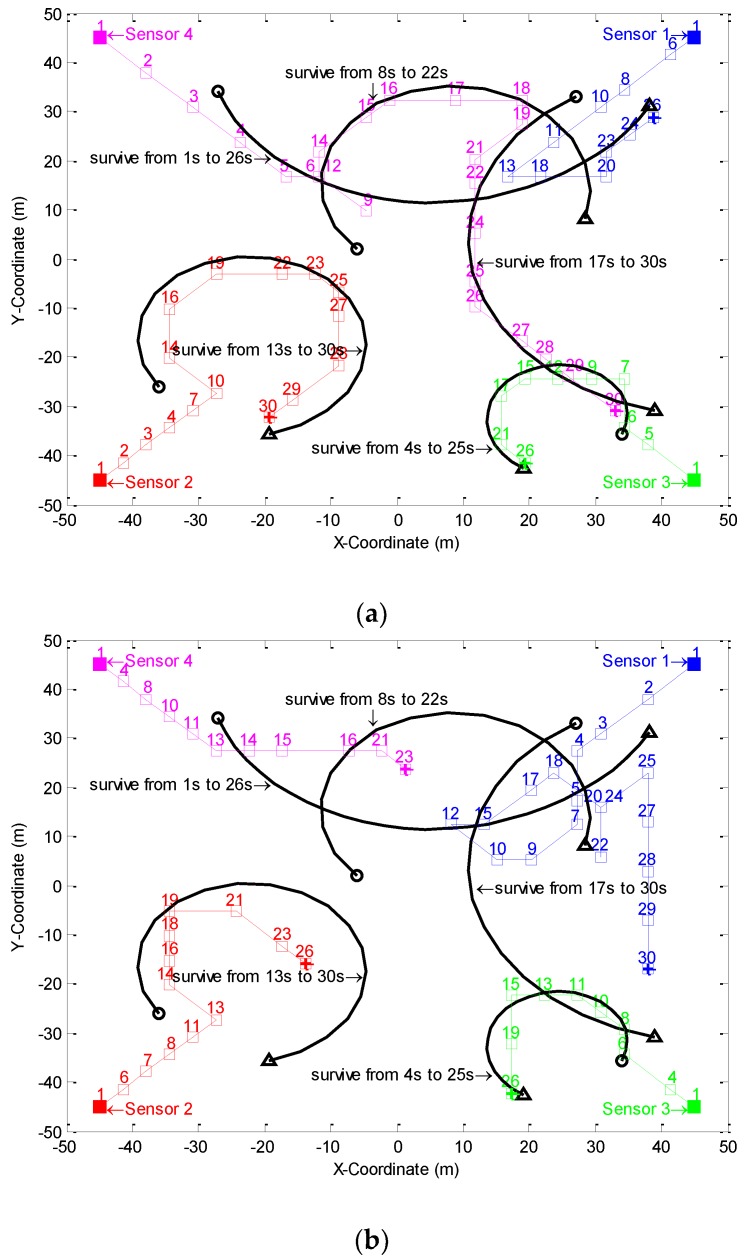
Sensor trajectories in a simulation by using (**a**) CS divergence with exhaustive search and (**b**) error bound with exhaustive search. The black line is the target trajectory, ○ and Δ are the target starting point and ending point respectively; The color line is the sensor trajectory, □ and the number above it are the sensor position and the time when the sensor is located at the position respectively, ■ and 田 are the sensor starting point and ending point respectively.

**Figure 2:**
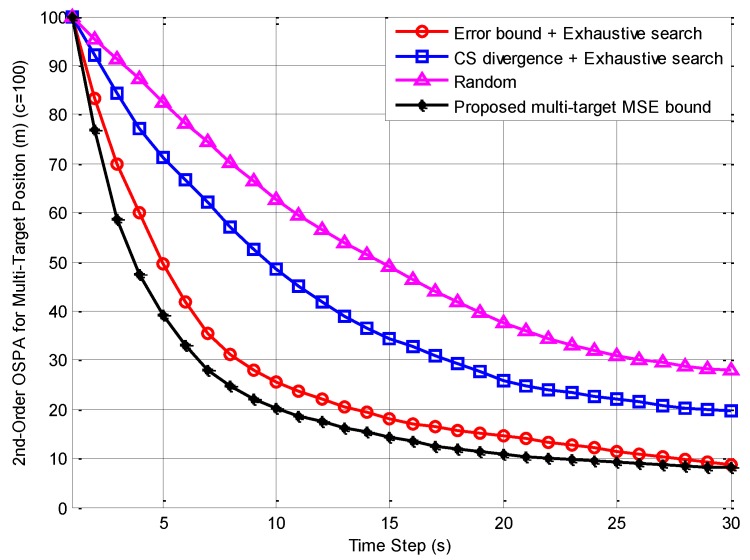
200 Monte Carlo (MC) run average of optimal sub-pattern assignment (OSPA) error distance and proposed mean-square error (MSE) bound for multi-target position estimates versus time.

Only the first two algorithms, which are the CS divergence with exhaustive search and the error bound with exhaustive search, were compared with the random control method in Example 1. There were two purposes for this. First, the number of the sensors in Example 1 was small and so, the computation cost of the exhaustive search method for solving the constrained optimization in (15) was completely acceptable. Second, we expected to clearly and separately present and analyze the effect of different multi-sensor control strategies (i.e., CS divergence based, error bound based and randomization based) without the influence of suboptimal algorithms (i.e., MPF and complex methods). Therefore, the simulation results of the error bound with MPF and complex were not shown temporarily in Figure 1 and Figure 2 of this example, since both of them jointly reflected the influence of control strategies and suboptimal algorithms. It was more appropriate to present the results for both of them together with the results of other scenarios in Table 4 and Table 5 of Example 2. In fact, the subsequent results in Example 2 also indicated that when the number of sensors is relatively small, the performance of the error bound with MPF or complex is so close to that of the error bound with exhaustive search that the difference among them is almost indistinguishable.

Figure 1 shows the trajectories of the four sensors by using the CS divergence with exhaustive search and the error bound with exhaustive search in a simulation. It was obviously meaningless to show the sensor trajectories of random control method since the sensor locations were completely randomly specified in this method.

Figure 2 shows the 200 MC run average of the OSPA error distance for multi-target position estimates versus time by using the methods of CS divergence with exhaustive search, error bound with exhaustive search, and random control. Furthermore, the proposed multi-target MSE bound is also presented in Figure 2 as an online performance indication for the multi-sensor control algorithms.

Figure 1 firstly shows that the methods of CS divergence and error bound with exhaustive search were both able to make the sensors move close to the corresponding survival targets. For the former, the motion distance of Sensor 4 was the longest (a total of 180 m) and the motion distance of Sensor 1 was the shortest (a total of 70 m). On the contrary, for the latter, the motion distance of Sensor 1 was the longest (a total of 165 m) and the motion distance of Sensor 4 was the shortest (a total of 55 m).

It can be seen from Figure 2 that among the three multi-sensor control algorithms, the averaged OSPA error distance from the error bound with exhaustive search was the smallest (finally about 8.7 m) and closest to the multi-target MSE bound. Besides, it had the fastest descent rate (50% reduction needs about 5 s); the averaged OSPA error distance from random control was the largest (finally about 28 m) and had the slowest descent rate (50% reduction needs about 15 s); the averaged OSPA error distance from CS divergence with exhaustive search and its descent rate were in the middle (finally about 19.5 m and 50% reduction needs about 10 s). This indicates that the method of the error bound with exhaustive search had the best multi-sensor control ability, which meant that its multi-target estimation accuracy was the highest. The random control method was the worst and the method of the CS divergence with exhaustive search was in the middle. The main reasons for the above results are:

(1) The GCI weights in the CS divergence with exhaustive search were set as the same, which indicates that each sensor had the same effect on the objective function (that is, the multi-sensor multi-target CS divergence). Therefore, under a certain energy consumption constraint, Sensor 4 had the highest control priority, since its energy consumption per meter is the smallest. Finally, it moved the longest distance. Sensor 1 was exactly the opposite of Sensor 4.

(2) The error bound with exhaustive search avoided the GCI rule. It can be seen from Theorem 1 that the effect of the sensors on the objective function (that is, multi-sensor multi-target MSE bound) was closely related to their likelihood functions. Therefore, even though the motion of Sensor 1 consumed the most energy per meter, it still had the highest control priority because its likelihood function had the greatest effect on the proposed bound. Finally, it moved the longest distance. Sensor 4 was exactly the opposite of Sensor 1.

(3) Since the error bound with exhaustive search was more effective than the CS divergence with exhaustive search for the control of Sensor 1, it had the best multi-target estimation accuracy as shown in Figure 2. The random control did not have the ability to make the sensors move close to the survival targets, so its multi-target estimation accuracy was the worst. The small gap between the multi-target MSE bound and the OSPA error distance from the error bound with exhaustive search was generated probably because the necessary and sufficient conditions for the equality sign of the generalized information inequality to RFS observation were not satisfied.

Above all, the simulation results were consistent with the above-mentioned intuitive conclusion.

### 5.2. Example 2: Scenarios with a Large Number of Sensors

In this example, the sensors of Example 1 and the energy-constrained threshold E in (48) were increased by one to four times; that is, the sensor number s and threshold E became (s=8,E=200 J), (s=12,E=300 J), (s=16,E=400 J), and (s=20,E=500 J). In order to jointly consider the effects of control strategies and suboptimal algorithms, the methods of CS divergence and error bound with exhaustive search are compared with the methods of error bound with MPF and complex, in both the MTT accuracy and computation cost. Here the computation cost for a run of an algorithm was indicated by its CPU processing time. Table 4 and Table 5 respectively show the 200 MC run averages of the final OSPA error distance and CPU processing time for the four methods under every scenario (including Example 1). The corresponding multi-target MSE bound is also presented in the last line of Table 4.

It should be noted that it is meaningless to show the relevant results of random control method in Table 4 and Table 5. This is because Example 1 has clearly indicated that the random control method always had the worst MTT accuracy since it could not make the sensors move gradually close to the targets according to certain information-driven or task-driven objective functions, as other sensor control methods. Moreover, it is also obvious that the computation cost of the random control method was much smaller than the other methods since it did not need to calculate the sensor control commands from a designed optimization problem at all.

From Table 4, it can be seen that the OSPA error distances of all the four methods and the multi-target MSE bound decreased with the increase of the number of sensors. The error bound with exhaustive search was always the best and the closest to the proposed bound, while the CS divergence with exhaustive search was always the worst. Moreover, the OSPA error distances of the last two methods were basically close to that of the error bound with exhaustive search, though they were up about 15% from the latter when *s* = 16 and *s* = 20. The reason for this is: At first, it is clear that the dimensions of the control command $u^{1:s}$ and its space $U^{1:s}$ increase with the sensor number *s*. The converged *s*-tuple control command obtained by the MPF method and complex method for the constrained optimization of (15) may be one of local optima. Given the objective function and constraints of the optimization, the number of the local optima increases with the dimension of the optimized control command $u^{1:s}$ [35,36]. Assuming that there are a total of M local optima and one of them is actual the global optimum, in the worst case the probability that the MPF or complex method converges to the global optimum will be 1/M. Therefore, although the methods proposed in [35,36,39] can more or less improve the chance of successfully converging to the global optimum for the MPF and complex methods, it is still inevitable that the solutions of multi-sensor control commands $u^{1:s}$ from the two suboptimal algorithms are more likely to fall into local optima when the sensor number *s* becomes larger. However, when the number of sensors has been relatively large (i.e., *s* = 16 and *s* = 20), the improvement of the estimation accuracy only by increasing the new sensors becomes very limited for all the four methods, and the gaps of their OSPA error distances become very small, too. On this occasion, it can be understood that the improvement of MTT accuracy reaches a ‘saturated’ state as the increase of the number of sensors.

From Table 5, it can be seen that with an increase in the number of sensors, the CPU processing times of the two exhaustive-search based methods were almost the same (their gap is less than 5% at every scenario), and their growth rates were significantly faster than those of the last two methods. When the number of sensors was relatively small (i.e., *s* = 4 and *s* = 8), the error bound with complex was the fastest and the following was the error bound with MPF. However, compared with the two exhaustive-search based methods, they did not accelerate enough. On the contrary, when the number of sensors was relatively large (i.e., *s* = 12, *s* = 16 and *s* = 20), the two suboptimal methods are significantly faster than the two exhaustive-search based methods. Furthermore, the error bound with MPF is faster than the error bound with complex in the case of a large number of sensors (i.e., the former was about 55% faster than the latter when *s* = 20). This is because that the iteration efficiency of the complex method becomes lower and so, its convergence rate becomes slower due to the high dimension of the optimized multi-sensor control command vector $\vec{u}={\left[{\left[u^{1}\right]}^{T},\ldots ,{\left[u^{s}\right]}^{T}\right]}^{T}$.

## 6. Conclusions and Future Work

Within the labeled RFS framework, a new constrained multi-sensor control algorithm is proposed for improving the performance of MTT. In this method, the multi-target MSE bound is treated as the cost function of the multi-sensor control and the control commands are just the optimal solution of the constrained optimization problem. In order to obtain the bound by using the generalized information inequality to RFS observation, the error between multi-target state set and its estimation is defined by the second-order OSPA metric, and the multi-target Bayes recursion is performed by the δ-GLMB filter. For the purpose of reducing computation cost, the MPF and complex methods are proposed to replace the exhaustive search method to solve the constrained optimization problem in the case of a large number of sensors. An advantage of our method is that it does not need to adopt the GCI fusion rule to obtain the multi-sensor posterior density from all the local posterior densities of every sensor, which avoids the possibility of improperly setting the normalized fusion weights in GCI. Simulation results show that for the constrained multi-sensor control system with the distinct observation performance, the multi-target estimation accuracy of our method is better than that of the GCI-based CS divergence method. Compared with the exhaustive search method in the case of a large number of sensors, the proposed MPF and complex methods can obviously reduce the computation time of finding the control commands from the constrained optimization problem at the expense of a little loss of estimation precision.

The future work will focus on the following five aspects:

(1) In current version of this paper, we implicitly assume that the communication capability meets the requirements of the proposed algorithms, and so the influences of communication range and bandwidth are not considered temporarily. For the sensor control problem, the limitations of communication range and bandwidth can be modeled as the relevant constraint conditions for the optimization of the control command. Therefore, one of our future works is to extend the proposed multi-sensor control method to the scenarios with communication constraints.

(2) When the number of sensors is very large, it is necessary to study the joint multi-sensor selection and control methods due to the limitations of the computation cost, energy consumption, communication range, and bandwidth, etc.

(3) It can be seen from [14] that PMBM filter has a strong resemblance to track-oriented multiple hypothesis tracking (MHT) method [42]. In fact, just for sharing information, this family of PMBM densities has the benefit that for each track hypothesis there is a Bernoulli component rather than a target that either exists or not as in δ-GLMB density. Because of this, one of these PMBM hypotheses can represent many δ-GLMB hypotheses with a corresponding increase in performance [15]. As a result, it is very helpful to improve the performance of multi-sensor control by replacing the δ-GLMB filter with the PMBM filter.

(4) Although the OSPA is currently the most popular metric in MTT, it does not penalize false targets, missed detections and localization errors, which are the usual/intuitive errors of interest in multitarget estimation. To solve this, a new improved metric called generalized OSPA (GOSPA) is proposed in [43]. Therefore, one of our research plans is to extend the multi-sensor control method of this paper based on the GOSPA metric because of its excellent consistency in mathematics and intuition compared with the OSPA metric.

(5) Recently, the distributed sensor network has been developed rapidly because of its advantages in scalability, flexibility, reliability, and ability of parallel computation. Therefore, it is very valuable and urgent to extend the multi-sensor control method of this paper to the distributed processing architecture.

## Figures and Tables

**Table 1:** Recursion for constrained multi-sensor control and multi-target tracking (MTT).

**Step** **1:** For each $u^{1:s}\in U_{D}^{1:s}$, calculate ${\underline{\sigma }}^{2}\left(u^{1:s}\right)$: ◆ Generate M samples $\Psi_{S}=\left\{{\tilde{X}}_{+}^{\left(1\right)},\ldots ,{\tilde{X}}_{+}^{\left(M\right)}\right\}$ according to the predicted multi-target δ-generalized labeled the multi-Bernoulli (δ-GLMB) density $\pi_{+}(X)$ in (17); ◆ For each ${\tilde{X}}_{+}^{\left(j\right)}\in \Psi_{S}$, generate predicted ideal measurement sets (PIMS) ${\tilde{Z}}_{m^{1:s}}^{1:s,(j)}$ of the s sensors according to the likelihood $g^{i}\left(\cdot |{\tilde{X}}_{+}^{\left(j\right)},u^{i}\right)$ in (7), i=1,…,s; ◆ For j=1,…,M, assign ${\tilde{Z}}_{m^{1:s}}^{1:s,(j)}$ to one of the measurement subspaces ${\mathbb{Z}}_{0,m^{1:s}}^{1:s},{\mathbb{Z}}_{1,m^{1:s}}^{1:s},\ldots ,{\mathbb{Z}}_{\infty ,m^{1:s}}^{1:s}$ according to (18) and (19); ◆ For $\hat{n}=0,\ldots ,\infty$, given the PIMS assigned to ${\mathbb{Z}}_{\hat{n},m^{1:s}}^{1:s}$, $\varpi_{\hat{n},n,m^{1:s}}\left(u^{1:s}\right)$ and $J_{\hat{n},n,m^{1:s}}\left(u^{1:s}\right)$ can be obtained by applying quasi MC method to (24) and (35); ◆ calculate $\Omega_{n,m^{1:s}}\left(u^{1:s}\right)$, $\Phi_{\hat{n},n,m^{1:s}}\left(u^{1:s}\right)$ and $\varepsilon_{\hat{n},n}$ according to (23), (33) and (34); ◆ ${\underline{\sigma }}^{2}\left(u^{1:s}\right)$ is obtained by substituting the calculated results of $\varpi_{\hat{n},n,m^{1:s}}\left(u^{1:s}\right)$, $\Omega_{n,m^{1:s}}\left(u^{1:s}\right)$, $\varepsilon_{\hat{n},n}$ and $\Phi_{\hat{n},n,m^{1:s}}\left(u^{1:s}\right)$ into (32); **Step** **2:** Calculate the optimal multi-sensor control commands ${\left[u^{1:s}\right]}^{*}$ according to (15); **Step** **3:** The real measurement sets $Z^{1:s}$ are received by the s sensors after executing the control commands ${\left[u^{1:s}\right]}^{*}$ **Step** **4:** The posterior multi-target δ-GLMB density $\pi \left(X|Z^{1:s},u^{1:s}\right)$ is obtained by substituting $Z^{1:s}$ into the update step of δ-GLMB filter; **Step** **5:** Extract the estimated set ${\hat{X}}_{\hat{n}}$ of multi-target states from $\pi \left(X|Z^{1:s},u^{1:s}\right)$ according to Assumption 3; **Step** **6:** Given that $\pi \left(X|Z^{1:s},u^{1:s}\right)$, the predicted multi-target δ-GLMB density $\pi_{+}(X)$ at the next time is obtained according to the prediction step of δ-GLMB filter; go to Step 1.

**Table 2:** Mixed penalty function (MPF) method for constrained multi-sensor control.

**Step** **1:** Select an initial control command $u_{\left(0\right)}^{1:s}$ that satisfies inequality constraints, initial barrier factor r, and reduction coefficient 0<C<1, set i=0. **Step** **2:** For j=1,…,s, calculate $u_{\left(i+1\right)}^{j}=\underset{u^{j}}{\arg\min}F\left(u_{\left(i+1\right)}^{1},\ldots ,u_{\left(i+1\right)}^{j-1},u^{j},u_{\left(i\right)}^{j+1},\ldots ,u_{\left(i\right)}^{s},r\right)$, where only $u^{j}$ is the variable and the others are treated as constants. **Step** **3:** If $\sum_{j=1}^{s}\left\|u_{\left(i+1\right)}^{j}-u_{\left(i\right)}^{j}\right\|\leq \varepsilon_{2}$, then go to Step 4, otherwise set i=i+1, go to Step 2. **Step** **4:** If $\sum_{j=1}^{s}\left\|u_{\left(i+1\right)}^{j}-u_{\left(0\right)}^{j}\right\|\leq \varepsilon_{1}$, then $u_{\left(i+1\right)}^{1:s}$ is outputted as the solution of (15), otherwise set r=Cr, $u_{\left(0\right)}^{1:s}=u_{\left(i+1\right)}^{1:s}$, i=0 and go to Step 2.

**Table 3:** Complex method for constrained multi-sensor control.

**Step** **1:** ${\vec{u}}_{1},\ldots ,{\vec{u}}_{k}\in {\vec{U}}_{D}$ (N+2≤k≤2N) as the k vertexes of initial complex. **Step** **2:** Find the worst vertex ${\vec{u}}_{W}$ which satisfies ${\underline{\sigma }}^{2}\left({\vec{u}}_{W}\right)=\max\left\{{\underline{\sigma }}^{2}\left({\vec{u}}_{1}\right),\ldots ,{\underline{\sigma }}^{2}\left({\vec{u}}_{k}\right)\right\}$ and the best vertex ${\vec{u}}_{B}$ which satisfies ${\underline{\sigma }}^{2}\left({\vec{u}}_{B}\right)=\min\left\{{\underline{\sigma }}^{2}\left({\vec{u}}_{1}\right),\ldots ,{\underline{\sigma }}^{2}\left({\vec{u}}_{k}\right)\right\}$ from the k vertexes. **Step** **3:** Calculate the center vertex ${\vec{u}}_{C}$ by use of the residual k−1 vertexes after excluding ${\vec{u}}_{W}$, ${\vec{u}}_{C}=\frac{1}{k-1}\left(\sum_{i=1}^{k}{\vec{u}}_{i}-{\vec{u}}_{W}\right)$. If ${\vec{u}}_{C}\in {\vec{U}}_{D}$, then go to Step 4; otherwise go to Step 1 to reselect the new initial vertexes. **Step** **4:** Calculate the reflecting vertex ${\vec{u}}_{R}$ of ${\vec{u}}_{W}$ with ${\vec{u}}_{C}$ as the axis, ${\vec{u}}_{R}={\vec{u}}_{C}+t\left({\vec{u}}_{C}-{\vec{u}}_{W}\right)$, where t>0 is a reflecting coefficient. If ${\vec{u}}_{R}\in {\vec{U}}_{D}$, then go to Step 5; otherwise set t=0.5t and repeat Step 4. **Step** **5:** If ${\underline{\sigma }}^{2}\left({\vec{u}}_{R}\right)<{\underline{\sigma }}^{2}\left({\vec{u}}_{W}\right)$, then set ${\vec{u}}_{W}={\vec{u}}_{R}$ and go to Step 6; otherwise set t=0.5t and go to Step 4. Note that if t has become very small (i.e., t is less than $10^{-5}$) but ${\underline{\sigma }}^{2}\left({\vec{u}}_{R}\right)<{\underline{\sigma }}^{2}\left({\vec{u}}_{W}\right)$ is still not satisfied, then it means that the reflecting direction formed by ${\vec{u}}_{W}$ and ${\vec{u}}_{C}$ is inappropriate. In order to change the reflecting direction, set ${\vec{u}}_{W}={\vec{u}}_{SW}$ and go to Step 3, where ${\vec{u}}_{SW}$ is the second-worse vertex. **Step** **6:** If ${\left(\frac{1}{k}\sum_{i=1}^{k}{\left[{\underline{\sigma }}^{2}\left({\vec{u}}_{C}\right)-{\underline{\sigma }}^{2}\left({\vec{u}}_{i}\right)\right]}^{2}\right)}^{\frac{1}{2}}\leq \varepsilon_{1}$ or $\underset{1\leq i\leq k}{\max}\left\|{\vec{u}}_{i}-{\vec{u}}_{C}\right\|\leq \varepsilon_{2}$, then ${\vec{u}}_{B}$ is output as the solution of (15), otherwise go to Step 2.

**Table 4:** 200 MC run averages of final optimal sub-pattern assignment (OSPA) error distance and multi-target MSE bound (Unit: m).

	Scenarios	*s* = 4, E = 100 J	*s* = 8, E = 200 J	*s* = 12, E = 300 J	*s* = 16, E = 400 J	*s* = 20, E = 500 J
Control Algorithms						
CS divergence with exhaustive search		19.5 m	13.9 m	9.2 m	5.8 m	4.6 m
Error bound with exhaustive search		8.7 m	6.0 m	4.6 m	3.9 m	3.6 m
Error bound with MPF		8.9 m	6.3m	5.1 m	4.5 m	4.1 m
Error bound with complex		9.0 m	6.4 m	5.1 m	4.4 m	4.0 m
Multi-target MSE bound		8.0 m	5.4 m	4.1 m	3.5 m	3.2 m

**Table 5:** 200 MC run averages of computer processing unit (CPU) processing time (Unit: s).

	Scenarios	*s* = 4, E = 100 J	*s* = 8, E = 200 J	*s* = 12, E = 300 J	*s* = 16, E = 400 J	*s* = 20, E = 500 J
Control Algorithms						
CS divergence with exhaustive search		22 s	153 s	1062 s	7259 s	50973 s
Error bound with exhaustive search		23 s	162 s	1128 s	7698 s	53168 s
Error bound with MPF		20 s	91 s	204 s	365 s	578 s
Error bound with complex		16 s	81 s	206 s	573 s	1296 s

## Appendix A. Proof of Lemma 1

Using the conditional probability formula, the posterior probability $P\left(|X|=n|Z^{1:s},u^{1:s}\right)$ can be written as

(A1) $$P\left(|X|=n|Z^{1:s},u^{1:s}\right)=\frac{P\left(|X|=n,Z^{1:s}|u^{1:s}\right)}{P\left(Z^{1:s}|u^{1:s}\right)}$$

where the numerator $P\left(|X|=n,Z^{1:s}|u^{1:s}\right)$ is the joint probability of $\left(|X|=n,Z^{1:s}\right)$ conditioned on $u^{1:s}$. It can be obtained as

(A2) $$\begin{array}{ll} P\left(|X|=n,Z^{1:s}|u^{1:s}\right) & =\frac{1}{n!}\sum_{\ell_{1:n}\in L_{n}}\int_{X_{n}}f\left(X_{n},Z^{1:s}|u^{1:s}\right)dx_{1:n}\\ & =\frac{1}{n!}\sum_{\ell_{1:n}\in L_{n}}\int_{X_{n}}\prod_{i=1}^{s}g^{i}\left(Z^{i}|X_{n},u^{i}\right)\pi_{+}\left(X_{n}\right)dx_{1:n} \end{array}$$

Substituting (7) and (17) into (A2) and then integrating the result over the region $X_{n}$, we get

(A3) $$\begin{array}{ll} P\left(|X|=n,Z^{1:s}|u^{1:s}\right) & =\frac{1}{n!}\sum_{\ell_{1:n}\in L_{n}}\sum_{\xi \in \Xi }\Delta \left(X_{n}\right)\omega_{+}^{\left(I,\xi \right)}\sum_{\theta^{1:s}\in \Theta^{1:s}}{\langle p_{+}^{\left(\xi \right)}\left(\cdot ,\ell \right),\prod_{i=1}^{s}\delta_{{\left(\theta^{i}\right)}^{-1}(\{0:|Z^{i}|\})}(I)\psi_{Z^{i},u^{i}}^{i}\left(\cdot ,\ell ;\theta^{i}\right)\rangle }^{I}\\ & =\sum_{(I,\xi )\in F_{n}(L)\times \Xi }\omega_{+}^{\left(I,\xi \right)}\sum_{\theta^{1:s}\in \Theta^{1:s}}{\langle p_{+}^{\left(\xi \right)}\left(\cdot ,\ell \right),\prod_{i=1}^{s}\delta_{{\left(\theta^{i}\right)}^{-1}(\{0:|Z^{i}|\})}(I)\psi_{Z^{i},u^{i}}^{i}\left(\cdot ,\ell ;\theta^{i}\right)\rangle }^{I} \end{array}$$

where the last line is derived by Lemma 12 of [9], $\psi_{Z^{i},u^{i}}^{i}\left(x,\ell ;\theta^{i}\right)$ is given by (8).

Using the law of total probability, the denominator $P\left(Z^{1:s}|u^{1:s}\right)$ of (A1) is obtained as

(A4) $$\begin{array}{ll} P\left(Z^{1:s}|u^{1:s}\right) & =\sum_{n=0}^{\infty }P\left(|X|=n,Z^{1:s}|u^{1:s}\right)\\ & =\sum_{(I,\xi )\in F(L)\times \Xi }\omega_{+}^{\left(I,\xi \right)}\sum_{\theta^{1:s}\in \Theta^{1:s}}{\langle p_{+}^{\left(\xi \right)}\left(\cdot ,\ell \right),\prod_{i=1}^{s}\delta_{{\left(\theta^{i}\right)}^{-1}(\{0:|Z^{i}|\})}(I)\psi_{Z^{i},u^{i}}^{i}\left(\cdot ,\ell ;\theta^{i}\right)\rangle }^{I} \end{array}$$

MAP detector determines $|\hat{X}|=\hat{n}$ if

(A5) $$\hat{n}=\underset{n}{\arg\max}\left\{P\left(|X|=n|Z^{1:s},u^{1:s}\right)\right\}$$

where

(A6) $$P\left(|X|=n|Z^{1:s},u^{1:s}\right)=\sum_{(I,\xi )\in F_{n}(L)\times \Xi }\sum_{\theta^{1:s}\in \Theta^{1:s}}\frac{\omega_{+}^{\left(I,\xi \right)}{\langle p_{+}^{\left(\xi \right)}\left(\cdot ,\ell \right),\prod_{i=1}^{s}\delta_{{\left(\theta^{i}\right)}^{-1}(\{0:|Z^{i}|\})}(I)\psi_{Z^{i},u^{i}}^{i}\left(\cdot ,\ell ;\theta^{i}\right)\rangle }^{I}}{\sum_{(I,\xi )\in F(L)\times \Xi }\omega_{+}^{\left(I,\xi \right)}\sum_{\theta^{1:s}\in \Theta^{1:s}}{\langle p_{+}^{\left(\xi \right)}\left(\cdot ,\ell \right),\prod_{i=1}^{s}\delta_{{\left(\theta^{i}\right)}^{-1}(\{0:|Z^{i}|\})}(I)\psi_{Z^{i},u^{i}}^{i}\left(\cdot ,\ell ;\theta^{i}\right)\rangle }^{I}}$$

is obtained by substituting (A3) and (A4) into (A1).

To simplify (A6), let

(A7) $$\omega_{Z^{1:s},u^{1:s}}^{\left(\xi ,\theta^{1:s}\right)}(I)=\frac{\omega_{+}^{\left(I,\xi \right)}{\langle p_{+}^{\left(\xi \right)}\left(\cdot ,\ell \right),\prod_{i=1}^{s}\delta_{{\left(\theta^{i}\right)}^{-1}(\{0:|Z^{i}|\})}(I)\psi_{Z^{i},u^{i}}^{i}\left(\cdot ,\ell ;\theta^{i}\right)\rangle }^{I}}{\sum_{(I,\xi )\in F(L)\times \mathbb{Z}}\omega_{+}^{\left(I,\xi \right)}\sum_{\theta^{1:s}\in \Theta^{1:s}}{\langle p_{+}^{\left(\xi \right)}\left(\cdot ,\ell \right),\prod_{i=1}^{s}\delta_{{\left(\theta^{i}\right)}^{-1}(\{0:|Z^{i}|\})}(I)\psi_{Z^{i},u^{i}}^{i}\left(\cdot ,\ell ;\theta^{i}\right)\rangle }^{I}}$$

which is exactly (19). $\omega_{Z^{1:s},u^{1:s}}^{\left(\xi ,\theta^{1:s}\right)}(I)$ actually indicates the conditional probability that the track label set is I, the association maps up to the last time are ξ and the current association map is $\theta^{1:s}$ given $Z^{1:s}$.

By the use of $\omega_{Z^{1:s},u^{1:s}}^{\left(\xi ,\theta^{1:s}\right)}(I)$, (A6) can be rewritten as:

(A8) $$P\left(|X|=n|Z^{1:s},u^{1:s}\right)=\sum_{(I,\xi )\in F_{n}(L)\times \Xi }\sum_{\theta^{1:s}\in \Theta^{1:s}}\omega_{Z^{1:s},u^{1:s}}^{\left(\xi ,\theta^{1:s}\right)}(I)$$

Finally, (18) can be obtained by substituting (A8) into (A5). ☐

## Appendix B. Proof of Theorem 1

From (11) and (20), (14) can be written as

(A9) $$\sigma^{2}\left(u^{1:s}\right)=\sum_{m^{s}=0}^{\infty }\cdots \sum_{m^{1}=0}^{\infty }\sum_{n=0}^{\infty }\frac{\Omega_{n,m^{1:s}}\left(u^{1:s}\right)}{m^{1}!\cdots m^{s}!n!}\sum_{\ell_{1:n}\in L_{n}}\int_{{\mathbb{Z}}_{m^{s}}^{s}}\cdots \int_{{\mathbb{Z}}_{m^{1}}^{1}}\int_{X_{n}}q\left(X_{n},Z_{m^{1:s}}^{1:s}|u^{1:s}\right)e^{2}\left(X_{n},\hat{X}\right)dx_{1:n}dz_{1:m^{1}}^{1}\cdots dz_{1:m^{s}}^{s}$$

where $\Omega_{n,m^{1:s}}\left(u^{1:s}\right)$ is defined by (21) and finally obtained as (23).

Dividing the integral region ${\mathbb{Z}}_{m^{1:s}}^{1:s}$ of (A9) into ${\mathbb{Z}}_{0,m^{1:s}}^{1:s},{\mathbb{Z}}_{1,m^{1:s}}^{1:s},\ldots ,{\mathbb{Z}}_{\infty ,m^{1:s}}^{1:s}$ according to Lemma 1, $\sigma^{2}\left(u^{1:s}\right)$ can be rewritten as

(A10) $$\sigma^{2}\left(u^{1:s}\right)=\sum_{m^{s}=0}^{\infty }\cdots \sum_{m^{1}=0}^{\infty }\sum_{n=0}^{\infty }\frac{\Omega_{n,m^{1:s}}\left(u^{1:s}\right)}{m^{1}!\cdots m^{s}!n!}\sum_{\hat{n}=0}^{\infty }\sum_{\ell_{1:n}\in L_{n}}\int_{{\mathbb{Z}}_{\hat{n},m^{s}}^{s}}\cdots \int_{{\mathbb{Z}}_{\hat{n},m^{1}}^{1}}\int_{X_{n}}q\left(X_{n},Z_{m^{1:s}}^{1:s}|u^{1:s}\right)e^{2}\left(X_{n},{\hat{X}}_{\hat{n}}\right)dx_{1:n}dz_{1:m^{1}}^{1}\cdots dz_{1:m^{s}}^{s}$$

Substituting (16) into (A10), we get

(A11) $$\begin{array}{c} \sigma^{2}\left(u^{1:s}\right)=\sum_{m^{s}=0}^{\infty }\cdots \sum_{m^{1}=0}^{\infty }\sum_{n=0}^{\infty }\frac{\Omega_{n,m^{1:s}}\left(u^{1:s}\right)}{m^{1}!\cdots m^{s}!n!}\sum_{\hat{n}=0,n+\hat{n}>0}^{\infty }\sum_{\ell_{1:n}\in L_{n}}\frac{1}{\max(\hat{n},n)}\int_{{\mathbb{Z}}_{\hat{n},m^{s}}^{s}}\cdots \int_{{\mathbb{Z}}_{\hat{n},m^{1}}^{1}}\int_{X_{n}}q\left(X_{n},Z_{m^{1:s}}^{1:s}|u^{1:s}\right)\\ \cdot \left(\underset{\tau \in \Gamma_{\max(\hat{n},n)}}{\min}\sum_{i=1}^{\min(\hat{n},n)}\min\left(c^{2},{\left\|x_{i}-{\hat{x}}_{\tau_{i}}\right\|}^{2}\right)+c^{2}\mathrm{abs}\left(\hat{n}-n\right)\right)dx_{1:n}dz_{1:m^{1}}^{1}\cdots dz_{1:m^{s}}^{s} \end{array}$$

Given the set ${\hat{X}}_{\hat{n}}$ of multi-target state estimates, let

(A12) $$\tau^{*}\left(X_{n}\right)≜\underset{\tau \in \Gamma_{\max(\hat{n},n)}}{\arg\min}\left\{\sum_{i=1}^{\min(\hat{n},n)}\min\left(c^{2},{\left\|x_{i}-{\hat{x}}_{\tau_{i}}\right\|}^{2}\right)\right\}$$

be the permutation to minimize the objective function $\sum_{i=1}^{\min\left(\hat{n},n\right)}\min\left(c^{2},{\left\|x_{i}-{\hat{x}}_{\tau_{i}}\right\|}^{2}\right)$ in $\Gamma_{\max\left(\hat{n},n\right)}$. Obviously, $\tau^{*}\left(X_{n}\right)$ depends on the set $X_{n}$ of true multi-target states. Through the minimization of (A12), $\tau^{*}\left(X_{n}\right)$ actually denotes the optimal assignment of target estimates ${\hat{X}}_{\hat{n}}$ to true target states $X_{n}$ under the cut-off c.

By the use of the optimal permutation $\tau^{*}\left(X_{n}\right)$ defined in (A12), (A11) becomes

(A13) $$\begin{array}{l} \sigma^{2}\left(u^{1:s}\right)=\sum_{m^{s}=0}^{\infty }\cdots \sum_{m^{1}=0}^{\infty }\sum_{n=0}^{\infty }\sum_{\hat{n}=0,n+\hat{n}>0}^{\infty }\frac{\Omega_{n,m^{1:s}}\left(u^{1:s}\right)\varpi_{\hat{n},n,m^{1:s}}\left(u^{1:s}\right)}{m^{1}!\cdots m^{s}!n!\max(\hat{n},n)}\\ \cdot \left(\begin{array}{l} \sum_{i=1}^{\min(\hat{n},n)}\min\left(c^{2},\frac{1}{\varpi_{\hat{n},n,m^{1:s}}\left(u^{1:s}\right)}\sum_{\ell_{1:n}\in L_{n}}\int_{{\mathbb{Z}}_{\hat{n},m^{s}}^{s}}\cdots \int_{{\mathbb{Z}}_{\hat{n},m^{1}}^{1}}\int_{X_{n}}q\left(X_{n},Z_{m^{1:s}}^{1:s}|u^{1:s}\right){\left\|x_{i}-{\hat{x}}_{\tau_{i}^{*}(X_{n})}\right\|}^{2}dx_{1:n}dz_{1:m^{1}}^{1}\cdots dz_{1:m^{s}}^{s}\right)\\ +c^{2}\mathrm{abs}\left(\hat{n}-n\right) \end{array}\right) \end{array}$$

where $\varpi_{\hat{n},n,m^{1:s}}\left(u^{1:s}\right)$ is defined by (22) and finally obtained as (24).

Making use of the marginal density $q_{n}\left(x,Z_{m^{1:s}}^{1:s}|u^{1:s}\right)$ defined by (29), the integration term in (A13) can be rewritten as

(A14) $$\begin{array}{l} \sum_{\ell_{1:n}\in L_{n}}\int_{{\mathbb{Z}}_{\hat{n},m^{s}}^{s}}\cdots \int_{{\mathbb{Z}}_{\hat{n},m^{1}}^{1}}\int_{X_{n}}q\left(X_{n},Z_{m^{1:s}}^{1:s}|u^{1:s}\right){\left\|x_{i}-{\hat{x}}_{\tau_{i}^{*}(X_{n})}\right\|}^{2}dx_{1:n}dz_{1:m^{1}}^{1}\cdots dz_{1:m^{s}}^{s}\\ =\int_{{\mathbb{Z}}_{\hat{n},m^{s}}^{s}}\cdots \int_{{\mathbb{Z}}_{\hat{n},m^{1}}^{1}}\int_{X_{1}}q_{n}\left(x,Z_{m^{1:s}}^{1:s}|u^{1:s}\right)\sum_{l=1}^{L}{\left(x^{l}-{\hat{x}}_{\tau_{i}^{*}(X_{n})}^{l}\right)}^{2}dxdz_{1:m^{1}}^{1}\cdots dz_{1:m^{s}}^{s} \end{array}$$

where L is the dimension of the unlabeled state x, $q_{n}\left(x,Z_{m^{1:s}}^{1:s}|u^{1:s}\right)=\sum_{\ell \in L_{1}}q_{n}\left(x,\ell ,Z_{m^{1:s}}^{1:s}|u^{1:s}\right)$ is the unlabeled version of (31).

Given the unbiased estimation criterion in Assumption 3, the generalized information inequality to RFS observation in (4) can be applied to the density $q_{n}\left(x,Z_{m^{1:s}}^{1:s}|u^{1:s}\right)$,

(A15) $$\int_{{\mathbb{Z}}_{\hat{n},m^{s}}^{s}}\cdots \int_{{\mathbb{Z}}_{\hat{n},m^{1}}^{1}}\int_{X_{1}}q_{n}\left(x,Z_{m^{1:s}}^{1:s}|u^{1:s}\right){\left(x^{l}-{\hat{x}}_{\tau_{i}^{*}(X_{n})}^{l}\right)}^{2}dxdz_{1:m^{1}}^{1}\cdots dz_{1:m^{s}}^{s}\begin{array}{cc} \geq {\left[J_{\hat{n},n,m^{1:s}}^{-1}\left(u^{1:s}\right)\right]}^{l,l} & l=1,\ldots ,L \end{array}$$

where $J_{\hat{n},n,m^{1:s}}\left(u^{1:s}\right)$ is the FIM with respect to the density $q_{n}\left(x,Z_{m^{1:s}}^{1:s}|u^{1:s}\right)$. $J_{\hat{n},n,m^{1:s}}\left(u^{1:s}\right)$ is obtained as (35). (A15) is satisfied with equality if and only if the density $q_{n}\left(x,Z_{m^{1:s}}^{1:s}|u^{1:s}\right)$ obeys a distribution of exponential family.

Note that as the optimal permutation $\tau^{*}\left(X_{n}\right)$ depends on true target states $X_{n}$, one cannot perform the integration on the left-hand side of the inequality (A15) as if it were independent on $X_{n}$. However, the lower bound ${\left[J_{\hat{n},n,m^{1:s}}^{-1}\left(u^{1:s}\right)\right]}^{l,l}$ on the integration can be obtained exactly according to the information inequality in (4). In addition, it can also be seen clearly from (35) that the FIM $J_{\hat{n},n,m^{1:s}}\left(u^{1:s}\right)$ in (A15) is independent of the specific state estimates ${\hat{X}}_{\hat{n}}$ and the optimal permutation $\tau^{*}\left(X_{n}\right)$ since $J_{\hat{n},n,m^{1:s}}\left(u^{1:s}\right)$ is completely determined by the density $q_{n}\left(x,Z_{m^{1:s}}^{1:s}|u^{1:s}\right)$ and its integral region $X_{1}\times {\mathbb{Z}}_{\hat{n},m^{1}}^{1}\times \cdots \times {\mathbb{Z}}_{\hat{n},m^{s}}^{s}$. As a result, for the various state estimate $\hat{x}$ and permutation τ in $\Gamma_{\max(\hat{n},n)}$, although the value of the integration $\int_{{\mathbb{Z}}_{\hat{n},m^{s}}^{s}}\cdots \int_{{\mathbb{Z}}_{\hat{n},m^{1}}^{1}}\int_{X_{1}}q_{n}\left(x,Z_{m^{1:s}}^{1:s}|u^{1:s}\right){\left(x^{l}-{\hat{x}}_{\tau_{i}}^{l}\right)}^{2}dxdz_{1:m^{1}}^{1}\cdots dz_{1:m^{s}}^{s}$ may change, the MSE lower bound on the unbiased estimate of x is always the same as $J_{\hat{n},n,m^{1:s}}^{-1}\left(u^{1:s}\right)$. Furthermore, it can be known from the references [44,45,46,47,48,49,50] that the integration on the left-hand side of the inequality (A15) will achieve the minimum value and so, be closest to the lower bound ${\left[J_{\hat{n},n,m^{1:s}}^{-1}\left(u^{1:s}\right)\right]}^{l,l}$ if the unbiased estimator $\hat{x}$ in (A15) is a Minimum Mean OSPA (MMOSPA) estimator.

Substituting (A15) into (A14) and then (A13), we get

(A16) $$\begin{array}{ll} \sigma^{2}\left(u^{1:s}\right)\geq & \sum_{m^{s}=0}^{\infty }\cdots \sum_{m^{1}=0}^{\infty }\sum_{n=0}^{\infty }\sum_{\hat{n}=0,n+\hat{n}>0}^{\infty }\frac{\Omega_{n,m^{1:s}}\left(u^{1:s}\right)\varpi_{\hat{n},n,m^{1:s}}\left(u^{1:s}\right)}{m^{1}!\cdots m^{s}!n!\max(\hat{n},n)}\\ & \cdot \left(\sum_{i=1}^{\min(\hat{n},n)}\min\left(c^{2},\frac{1}{\varpi_{\hat{n},n,m^{1:s}}\left(u^{1:s}\right)}\sum_{l=1}^{L}{\left[J_{\hat{n},n,m^{1:s}}^{-1}\left(u^{1:s}\right)\right]}^{l,l}\right)+c^{2}\mathrm{abs}\left(\hat{n}-n\right)\right) \end{array}$$

Since both of $J_{\hat{n},n,m^{1:s}}\left(u^{1:s}\right)$ and $\varpi_{\hat{n},n,m^{1:s}}\left(u^{1:s}\right)$ are clearly independent of the $i=1,\ldots ,\min(\hat{n},n)$, (A16) can be reduced to

(A17) $$\begin{array}{ll} \sigma^{2}\left(u^{1:s}\right)\geq & \sum_{m^{s}=0}^{\infty }\cdots \sum_{m^{1}=0}^{\infty }\sum_{n=0}^{\infty }\sum_{\hat{n}=0,n+\hat{n}>0}^{\infty }\frac{\Omega_{n,m^{1:s}}\left(u^{1:s}\right)\varpi_{\hat{n},n,m^{1:s}}\left(u^{1:s}\right)}{m^{1}!\cdots m^{s}!n!\max(\hat{n},n)}\\ & \cdot \left(\min(\hat{n},n)\cdot \min\left(c^{2},\frac{1}{\varpi_{\hat{n},n,m^{1:s}}\left(u^{1:s}\right)}\sum_{l=1}^{L}{\left[J_{\hat{n},n,m^{1:s}}^{-1}\left(u^{1:s}\right)\right]}^{l,l}\right)+c^{2}\mathrm{abs}\left(\hat{n}-n\right)\right) \end{array}$$

Finally, (32)–(34) can easily be obtained from (A17).

One more important thing to be explained in detail is the relationship between the proposed bound of Theorem 1 and the Minimum Mean OSPA (MMOSPA) presented in [44,45,46,47,48,49,50]: It can be known from the references [44,45,46,47,48,49,50] that the proposed OSPA-based multi-target MSE defined in (14) of this paper is actually the Mean OSPA (MOSPA) in [44,45,46,47,48,49,50]. The MOSPA achieves the minimum value when the estimator $\hat{X}$ is a MMOSPA estimator. However, even for the MMOSPA estimator, the information inequality in (A15) (and finally the proposed bound in (32)) still holds as long as the MMOSPA estimator satisfies the unbiased estimation criterion. In other words, when the estimator used in the information inequality of (4) is a MMOSPA estimator and is unbiased, the relevant MOSPA (also the multi-target MSE defined in (14)) is the minimum and thus, is closest to the lower bound presented in Theorem 1.
